# Rational Design and Biological Application of Antioxidant Nanozymes

**DOI:** 10.3389/fchem.2020.00831

**Published:** 2021-02-11

**Authors:** Ruizhen Tian, Jiayun Xu, Quan Luo, Chunxi Hou, Junqiu Liu

**Affiliations:** ^1^State Key Laboratory of Supramolecular Structure and Materials, College of Chemistry, Jilin University, Changchun, China; ^2^College of Material, Chemistry and Chemical Engineering, Hangzhou Normal University, Hangzhou, China

**Keywords:** antioxidants, nanozyme, oxidative stress, rational design, biological application

## Abstract

Nanozyme is a type of nanostructured material with intrinsic enzyme mimicking activity, which has been increasingly studied in the biological field. Compared with natural enzymes, nanozymes have many advantages, such as higher stability, higher design flexibility, and more economical production costs. Nanozymes can be used to mimic natural antioxidant enzymes to treat diseases caused by oxidative stress through reasonable design and modification. Oxidative stress is caused by imbalances in the production and elimination of reactive oxygen species (ROS) and reactive nitrogen species (RNS). This continuous oxidative stress can cause damage to some biomolecules and significant destruction to cell structure and function, leading to many physiological diseases. In this paper, the methods to improve the antioxidant properties of nanozymes were reviewed, and the applications of nanozyme antioxidant in the fields of anti-aging, cell protection, anti-inflammation, wound repair, cancer, traumatic brain injury, and nervous system diseases were introduced. Finally, the future challenges and prospects of nanozyme as an ideal antioxidant were discussed.

## Introduction

Reactive oxygen species (ROS) are produced in the normal physiological activities of aerobic organisms. Oxygen (O_2_) undergoes a series of electron transport in biological metabolism, which results in the formation of ROS, such as superoxide anion radical (${\text{O}}_{2}^{•-}$), hydrogen peroxide (H_2_O_2_), hydroxyl radicals (^•^OH) (Winterbourn, 2008; Dickinson and Chang, 2011; Nathan and Cunningham-Bussel, 2013). Appropriate amounts of ROS participate in a variety of signal pathways in response to changes in external conditions and play an essential role as a second messenger in signal transduction, immune response, and cell function regulation (Gechev et al., 2006; D'Autréaux and Toledano, 2007; Finkel, 2011; Sena and Chandel, 2012; Schieber and Chandel, 2014). There is an antioxidant system composed of non-enzymatic antioxidant molecules and natural antioxidant enzymes in the organism to maintain the balance of ROS. Some non-enzymatic antioxidants, such as ascorbic acid (AA), reductive glutathione (GSH), vitamin E (VE), and melanin, are effective in scavenging free radicals (Niki and Noguchi, 2004; Liu Y. L. et al., 2012; Kakaroubas et al., 2019). The natural synergistic antioxidant system mainly includes superoxide dismutase (SOD), glutathione peroxidase (GPx), catalase (CAT) (Morry et al., 2016). Nevertheless, too much ROS consumes a lot of antioxidant molecules and attacks antioxidant enzymes, which can cause oxidative stress when the redox homeostasis is disturbed (Griendling and FitzGerald, 2003; Reuter et al., 2010). This continuous oxidative stress can cause severe damage to some biomolecules, such as DNA, lipids, proteins, and significant destruction of cell structure and function (Ray et al., 2000; Valko et al., 2004; Dalle-Donne et al., 2006). Severe cell damage and tissue inflammation can also induce many physiological diseases (Nechifor et al., 2009) such as diabetes (Baynes, 1991), sepsis (Macdonald et al., 2003), atherosclerosis (Harrison et al., 2003), arthritis (Tak et al., 2000), aging (Vitale et al., 2013), kidney disease (Forbes et al., 2008), cardiovascular diseases (Cai and Harrison, 2000), nervous system diseases (Barnham et al., 2004), and lung diseases (Ceccarelli et al., 2008).

**Scheme 1:**
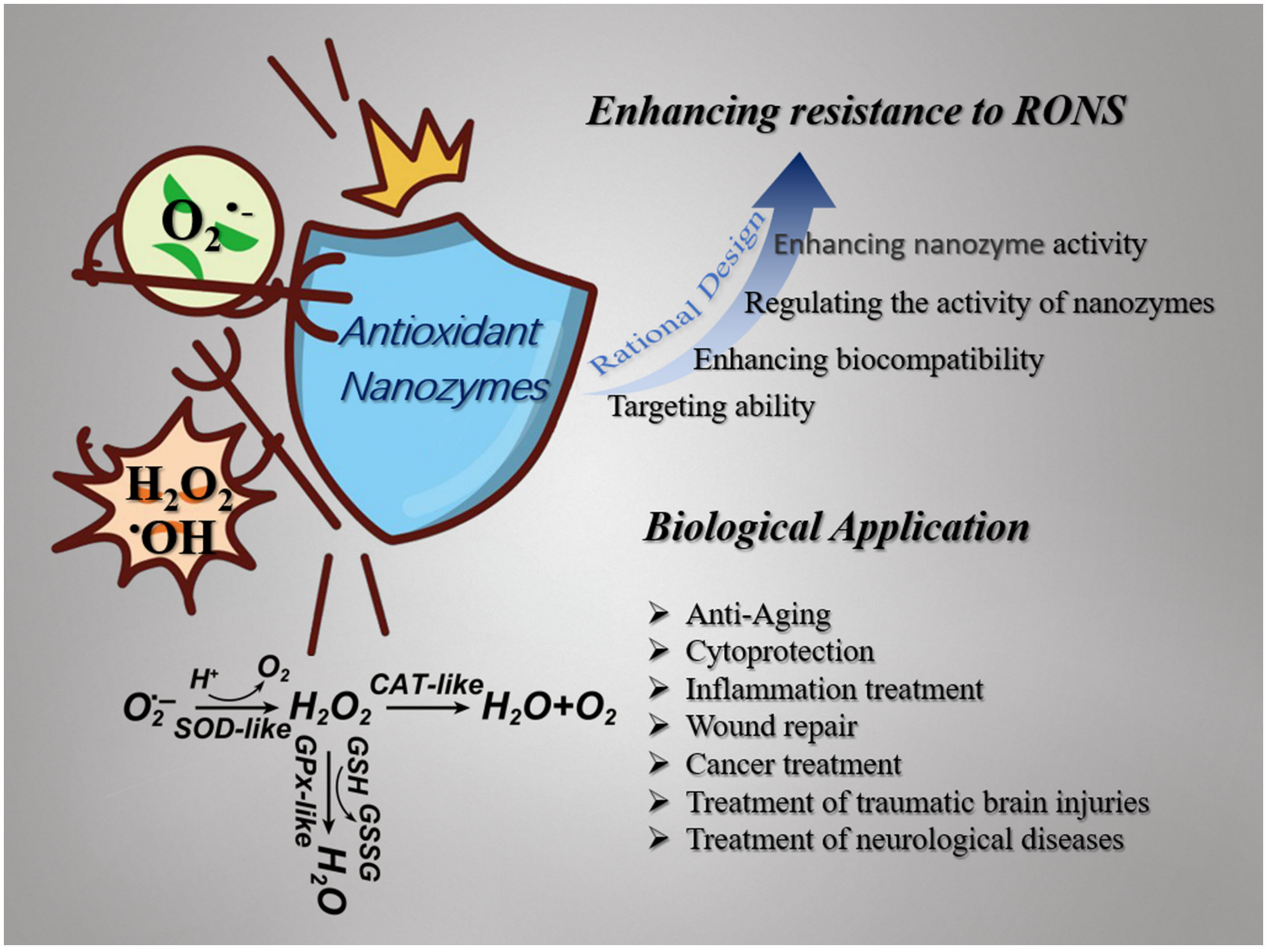
Schematic illustration of nanozymes with multienzyme-like activity (SOD-like, GPx-like, CAT-like, et al.) against ROS (${\text{O}}_{2}^{•-}$, H_2_O_2_, ^•^OH, et al.). Nanozymes are broadly used in the field of biomedicine as antioxidants and their antioxidant capacity can be enhanced by rational design.

Although a wide range of antioxidants has been widely used to inhibit and fight oxidative stress-related pathological diseases, there are still some severe limitations. For example, natural enzyme antioxidants lack stability and are readily inactivated under non-physiological conditions. Some non-enzymatic antioxidants cannot pass through the blood-brain barrier (Gilgun-Sherki et al., 2001) and have low bioavailability (Heim et al., 2002). Compared with traditional antioxidants, nanozyme antioxidant is a kind of enzyme mimetics based on nanomaterials, which has the characteristics of flexible operation, excellent stability, low cost, mass production, and easy treatment (Wu et al., 2019). In recent years, many different inorganic nanomaterials have been developed as antioxidant nanozymes, such as noble metals (Liu X. P. et al., 2016), metal oxides (Soh et al., 2017), carbon-based nanomaterials (Mu et al., 2019a), and other substrates (Zhao et al., 2018). The ability of nanozymes to scavenge ROS mainly originates from intrinsic SOD, CAT, GPx, NAC mimicking activities, or peroxidase (POD) mimicking activity without producing hydroxyl radicals, and ^•^OH-, ^•^DPPH-, or ^•^NO-scavenging activity (Akhtar et al., 2015b; Chen et al., 2018; Hao et al., 2019; Yan et al., 2019). Nanozymes are often accompanied by a variety of enzyme mimetic activities that can efficiently scavenge ROS, but there are also some inherent shortcomings. The toxicity of nanoparticles is the first consideration in biological applications. It has been shown that some inorganic nanoparticles can interact with lipid, proteins, and DNA, thereby impairing the integrity of biofilms and the function of enzymes (Cedervall et al., 2007; Wang et al., 2008; Pelka et al., 2009). The significant disadvantages of nanozymes are insufficient targeting and lacking the ability to bind to substrates specifically, which affect the effectiveness of the treatment of the disease. Some nanozymes have an antagonistic effect, which can catalyze the generation and elimination of ROS, which is not conducive to biological applications. The size and morphology of nanoparticles have a significant influence on the activity and function of mimetic enzymes. Therefore, it is necessary to rationally design the nanozyme to improve the biocompatibility, targeting, adjust, and enhance the activity of the nanozyme, reduce the dosage, and get a better application in the biological field. This review focuses on rationally designing nanozymes to enhance their antioxidant capacity and their application in biomedical field (Scheme 1).

## Antioxidant Enzyme Activities of Nanozymes

In this section, we introduce the antioxidant properties of nanozymes, which have been developed as antioxidants in recent years (Table 1).

**Table 1:** Summary of the applications of nanozymes as antioxidant in recent years.

**Nanozymes**	**Enzyme mimic activity**	**Biological application**	**Reference**
Porous CuxO NPs	SOD, CAT, and GPx-like	Parkinson's Disease	Hao et al., 2019
Porous platinum NPs	CAT-like	Enhance radiation efficacy	Li et al., 2019
Carbogenic nanozyme	SOD, CAT-like ^•^NO and ONOO^−^scavenging activities	Traumatic brain injury	Mu et al., 2019a
Pt/CeO_2_	POD, SOD, CAT, GPx-like, and ^•^OH and ONOO^−^ scavenging activities	Brain trauma	Yan et al., 2019
CuTA nanosheets	SOD-like, CAT-like, and ^•^OH scavenging activity	Smoking-induced lung destruction	Lin et al., 2019
A single-atom Fe–N_4_ catalytic site	SOD-like, CAT-like	Cytoprotection	Ma et al., 2019
PtPdMo trim	POD, CAT-like ^•^OH, ^1^O_2_, and ^•^NO scavenging activities	Brain injury	Mu et al., 2019b
Fe_3_O_4_ NPs	CAT-like	Anti-Aging Experimental cerebral malaria	Zhang et al., 2015 Zhao et al., 2019
MoS_2_ NPs	SOD, CAT-like	Osteoarthritis	Chen et al., 2019
CeO_2_ NPs	SOD-like, CAT-like	Parkinson's disease Neuroprotective effect, Regenerative wound healing	Kwon et al., 2018 Zeng et al., 2018 Wu et al., 2018
Prussian blue NPs	POD, CAT, and SOD-like	Reduce colitis in mice	Zhao et al., 2018
Silver-gold-apoferritin nanozyme	POD, CAT, and SOD-like	Suppress oxidative stress during cryopreservation	Dashtestani et al., 2018
MoS_2_ nanosheets	SOD, CAT, POD-like, and ^•^OH-, ^•^DPPH-, and ^•^NO- scavenging activity	Cytoprotection	Chen et al., 2018
Se@Pda	GPx	Anti-inflammation	Huang et al., 2018
Mn_3_O_4_ NPs	SOD, CAT, GPx-like, and ^•^OH scavenging activity	Anti-inflammation Parkinson's disease	Singh et al., 2017 Yao et al., 2018
Ce_0.7_Zr_0.3_O_2_ NPs	SOD, CAT-like, and ^•^OH scavenging activity	Anti-inflammation	Soh et al., 2017
GO-Se nanocomposite	GPx-like	Cytoprotection	Huang et al., 2017
Se-CQDs	^•^OH scavenging activity	Cytoprotection	Li F. et al., 2017
Melanin NPs	SOD-like	Ischemic stroke	Liu et al., 2017
ZnO/CeOx HMS	SOD-like, CAT-like	UV-induced epidermal hypertrophy	Ju et al., 2017
Multicomponent nanoreactor	Photosynthesizing H_2_ gas	Anti-inflammation	Wan et al., 2017
Ceria/POMs hybrid NPs	Proteolytic and SOD-like	Treatment of neurotoxicity of amyloid-β peptide	Guan et al., 2016
Pt NPs	SOD, CAT, POD-like	Human Cerebral Cavernous Malformation (CCM) disease	Moglianetti et al., 2016
MnO_2_ NPs	SOD, CAT-like	Overcome tumor hypoxia, Cytoprotection	Song et al., 2016 Li W. et al., 2017
V_2_O_5_@pDA@MnO_2_ nanocomposite	SOD, CAT, and GPx-like	Anti-inflammation	Huang et al., 2016
PVP-IrNPs	CAT, POD-like	Cytoprotection	Su et al., 2015
Molybdenum NPs (Mo NPs)	NAC-like	Human breast and fibrosarcoma cells	Akhtar et al., 2015a

**Figure 1:**
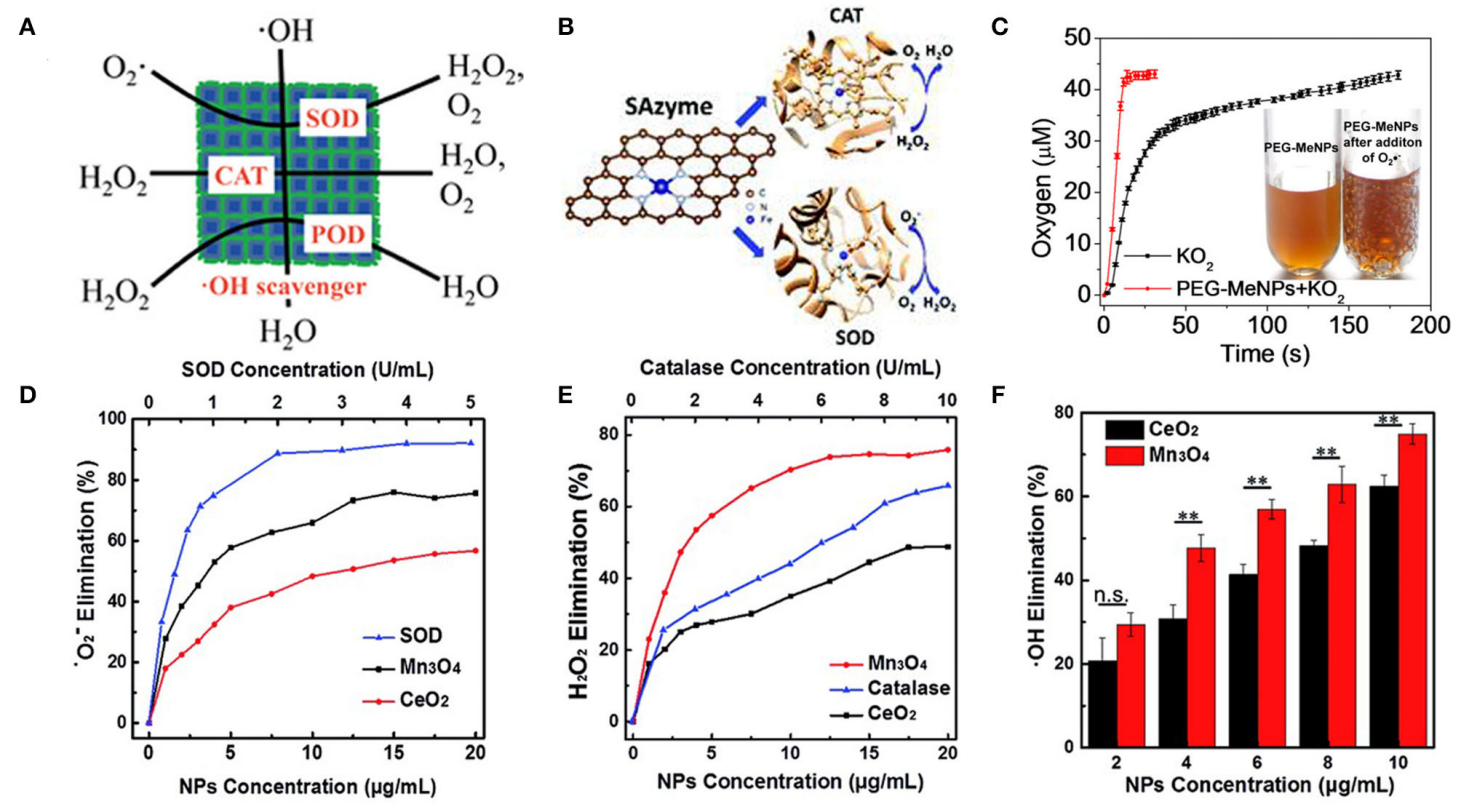
Antioxidant activity of nanozymes. **(A)** The PPBs could act as an artificial enzyme, effectively scavenging ROS including ^•^OH, H_2_O_2_, and ^•^OOH via POD, CAT, and SOD activity [reproduced from Zhao et al. (2018) with permission from the American Chemical Society]. **(B)** Atomically dispersed Fe–N_4_ sites anchored on N-doped porous carbon materials [reproduced from Ma et al. (2019) with permission from the Royal Society of Chemistry (RSC)]. **(C)** O_2_ production from the KO_2_ solution (100 μM) with vs. without PEG-MeNPs. The insert is the digital picture of the PEG-MeNPs solution before vs. after addition of KO_2_ [reproduced from Liu et al. (2017) with permission from the American Chemical Society]. **(D)** Dependence between the elimination of ^•^${\text{O}}_{2}^{-}$ and concentrations of Mn_3_O_4_ NPs, CeO_2_ NPs, and natural SOD. **(E)** Dependence between the elimination of H_2_O_2_ and concentrations of Mn_3_O_4_ NPs, CeO_2_ NPs, and natural catalase. **(F)** Dependence between the elimination of ^•^OH and concentration of Mn_3_O_4_ NPs and CeO_2_ NPs (mean ± S.D., **p < 0.05; n.s., not significant) [reproduced from Yao et al. (2018) with permission from the Royal Society of Chemistry (RSC)].

Prussian blue nanoparticles have also been found to have a variety of antioxidant enzyme activities, including peroxidase activity, catalase activity, and superoxide dismutase activity, which can effectively scavenge ROS (Figure 1A). Chen's group reported a polyvinylpyrrolidone (PVP)-modified Prussian blue nanoparticle (PPB) with good biological safety and physiological stability. The prepared PPBs have the abilities of scavenging ROS and inhibiting proinflammatory cytokines. The intravenous administration of PPBs can significantly reduce colitis without obvious side effects. A hydroxyl radical-generating TiO_2_/UV system was used to investigate the ROS scavenging ability of PPBs. As the concentration of PPBs increases from 0 to 10 μg/mL, the signal intensity of BMPO/^•^OH displays a steep decline, indicating the excellent scavenging capability of PPBs against ^•^OH. The effect of PPBs on ^•^OH can be represented in reactions:

$$\begin{array}{r} {\text{K}}_{\text{x}}\text{Fe}{\left(\text{III}\right)}_{\text{y}}{\left[\text{Fe}(\text{II}){\left(\text{CN}\right)}_{6}\right]}_{\text{z}}(\text{PB})\\ {\text{K}}_{\text{x}}\text{Fe}{\left(\text{III}\right)}_{\text{y}}{\left[\text{Fe}(\text{III}){\left(\text{CN}\right)}_{6}\right]}_{\text{n}}{\left[\text{Fe}(\text{II}){\left(\text{CN}\right)}_{6}\right]}_{\text{z}-\text{n}}(\text{BG})\\ {\text{K}}_{\text{x}}\text{Fe}{\left(\text{III}\right)}_{\text{y}}{\left[\text{Fe}(\text{III}){\left(\text{CN}\right)}_{6}\right]}_{\text{z}}(\text{PY}) \end{array}$$

(1) $$\text{PB}\to \text{BG}+{\text{e}}^{-}$$

(2) $$\text{BG}\to \text{PY}+(\text{z}-\text{n}){\text{e}}^{-}$$

(3) $$\text{PB}+{\text{H}}^{+}+HO\to \text{PY}+{\text{H}}_{2}\text{O}$$

The addition of H_2_O_2_ into the PPB solution generated a number of observable bubbles, catalyzing the decomposition of H_2_O_2_ to produce oxygen. The CAT-like activity of PPBs can be shown in the following reactions

(4) $$\text{PB}+{\text{H}}_{2}{\text{O}}_{2}\to \text{BG}+{\text{OH}}^{-}$$

(5) $$\text{BG}+{\text{H}}_{2}{\text{O}}_{2}\to \text{PY}+{\text{OH}}^{-}$$

(6) $$\text{PY}+{\text{H}}_{2}{\text{O}}_{2}+{\text{OH}}^{-}\to \text{BG}+{\text{O}}_{2}+{\text{H}}_{2}\text{O}$$

(7) $$\text{BG}+{\text{H}}_{2}{\text{O}}_{2}+{\text{OH}}^{-}\to \text{PB}+{\text{O}}_{2}+{\text{nH}}_{2}\text{O}$$

(Zhao et al., 2018).

In addition, the commonly used natural POD substrates 3,5,3,5-tetramethylbenzidine (TMB) were also determined to study the POD activity of PPBs. The absorbance increased after the addition of PPBs, indicative of POD-like activity.

(8) $$\text{TMB}+{\text{H}}_{2}{\text{O}}_{2}+{\text{H}}^{+}\overset{\text{PY}}{\to }{\text{TMB}}_{\left(\text{oxidized}\right)}+{\text{H}}_{2}\text{O}$$

The authors used the xanthine/xanthine oxidase system to investigate the effects of PPBs on superoxide radicals (^•^OOH). With the increase of PPBs concentration, the signal intensity of BMPO/^•^OOH decreased significantly, indicating that PPBs could be used as SOD-like nanozyme to scavenge ^•^OOH.

(9) O•OHSOD/PB→SOD/PBH2O2+O2

In conclusion, the PPBs can be used as artificial nanozymes to effectively convert harmful ROS to H_2_O and O_2_ and to avoid lipid peroxidation, protein oxidation and DNA damage (Zhao et al., 2018).

Li's group demonstrated that single-atom catalysts can be used as single-atom nanozymes (SAzymes) with multiple antioxidant activities (Figure 1B). Atom-dispersed Fe-N_4_ active sites can mimic SOD and CAT activities, effectively removing H_2_O_2_ and O_2._ To compare the efficiency of Fe-SAs/NC with other reported nanozymes, the authors calculated the TOF (turnover frequency) values per active site of Fe-SAs/NC-based enzyme and nanozymes. The TOF of Fe-SAs/NC was estimated to be 1809.34 min^−1^, which is much higher than other nanozymes such as Pd octahedrons (1.51 min^−1^) (Ge et al., 2016) and Mn_3_O_4_ nanoflowers (111.86 min^−1^) (Singh et al., 2017) reported previously (Ma et al., 2019).

Melanin nanoparticles modified PEG can provide more effective and safer antioxidant therapy. The authors speculated the mechanism of melanin scavenging ${\text{O}}_{2}^{•-}$, as follos:

$$\begin{array}{lll} {\text{melanin}}^{•} & + & {{\text{O}}_{2}}^{•-}\to {\text{melanin}}^{-}+\;{\text{O}}_{2}\\ {\text{melanin}}^{-} & + & {{\text{O}}_{2}}^{•-}+{2\text{H}}_{2}\text{O}\to {\text{melanin}}^{•}+{\text{H}}_{2}{\text{O}}_{2}+\;{2\text{OH}}^{-} \end{array}$$

When ${\text{O}}_{2}^{•-}$ was added, a large number of bubbles appeared in the PEG-MeNPs solution (Figure 1C). PEG-MeNPs could also scavenge ^•^OH and ONOO–, which are the most toxic secondary electrons produced in diseases and can cause lipid peroxidation, protein oxidation, and nucleic acid damage, and maintain high stability. Moreover, they found that the PEG-MeNPs blocked the formation of ^•^OH, possibly because melanin had a strong chelating ability with transition metal ions, which impeded the Fenton reaction (Liu et al., 2017).

CeO_2_ NPs are well-known as a superoxide dismutase (SOD) mimetic due to the redox cycle between Ce^3+^ and Ce^4+^, Ce^4+^ sites are responsible for the decomposition of hydrogen peroxide through the CAT-like activity (Korsvik et al., 2007; Pirmohamed et al., 2010). Mn_3_O_4_ NPs have been demonstrated to possess remarkable SOD-like activity (Figure 1D), due to the mixed valance states of Mn^2+^ and Mn^3+^. Besides, Mn_3_O_4_ has CAT-like activity (Figure 1E) and ^•^OH scavenging activity (Figure 1F). Compared with CeO_2_ NPs, Mn_3_O_4_ nanoparticles exhibit higher ROS scavenging capability (Figures 1D–F) (Yao et al., 2018). Mugesh's group demonstrated for the first time that Mn_3_O_4_ nanoparticles with flower-like morphology (Mnf) could mimic the activity of three antioxidant enzymes, including SOD, CAT, and GPx. The multienzyme activity of Mnf may be due to the existence of two oxidation states of manganese (Mn^2+^/Mn^3+^), large surface area, and abnormal large pore size. To understand the relative influence of the two different oxidation states on the activity of Mnf, the author treated Mnf with an oxidizing agent (NaIO_4_) and a reducing agent (NaBH_4_) to obtain the oxidized (O-Mnf) and reduced (R-Mnf) forms with different ratios of Mn^3+^/Mn^2+^. Interestingly, O-Mnf, with a higher Mn^3+^/Mn^2+^ ratio, exhibited enhanced CAT and GPx-like activities compared with Mnf. In contrast, the SOD activity of O-Mnf and R-Mnf was slightly higher than that of Mnf, suggesting that the oxidation states of both played a crucial role in enzyme mimetic activity. In the experimental model of Parkinson's disease, Mnf could be effectively internalized into human cells, inhibited apoptosis caused by the neurotoxin, and played an influential role in cytoprotection (Singh et al., 2017). Few-layer MoS_2_ nanosheets possessed intrinsic activity of mimicking SOD, CAT, and POD under physiological conditions (pH 7.4, 25°C). The POD-like activity originated from their ability to transfer electrons without producing hydroxyl radicals (Chen et al., 2018). Fullerene-Like MoS_2_ (F-MoS_2_) nanoparticles are effective lubricants and antioxidants for artificial synovial fluid due to their unique structures and intrinsic dual-enzyme-like (SOD- and CAT-like) activity (Chen et al., 2019).

**Figure 2:**
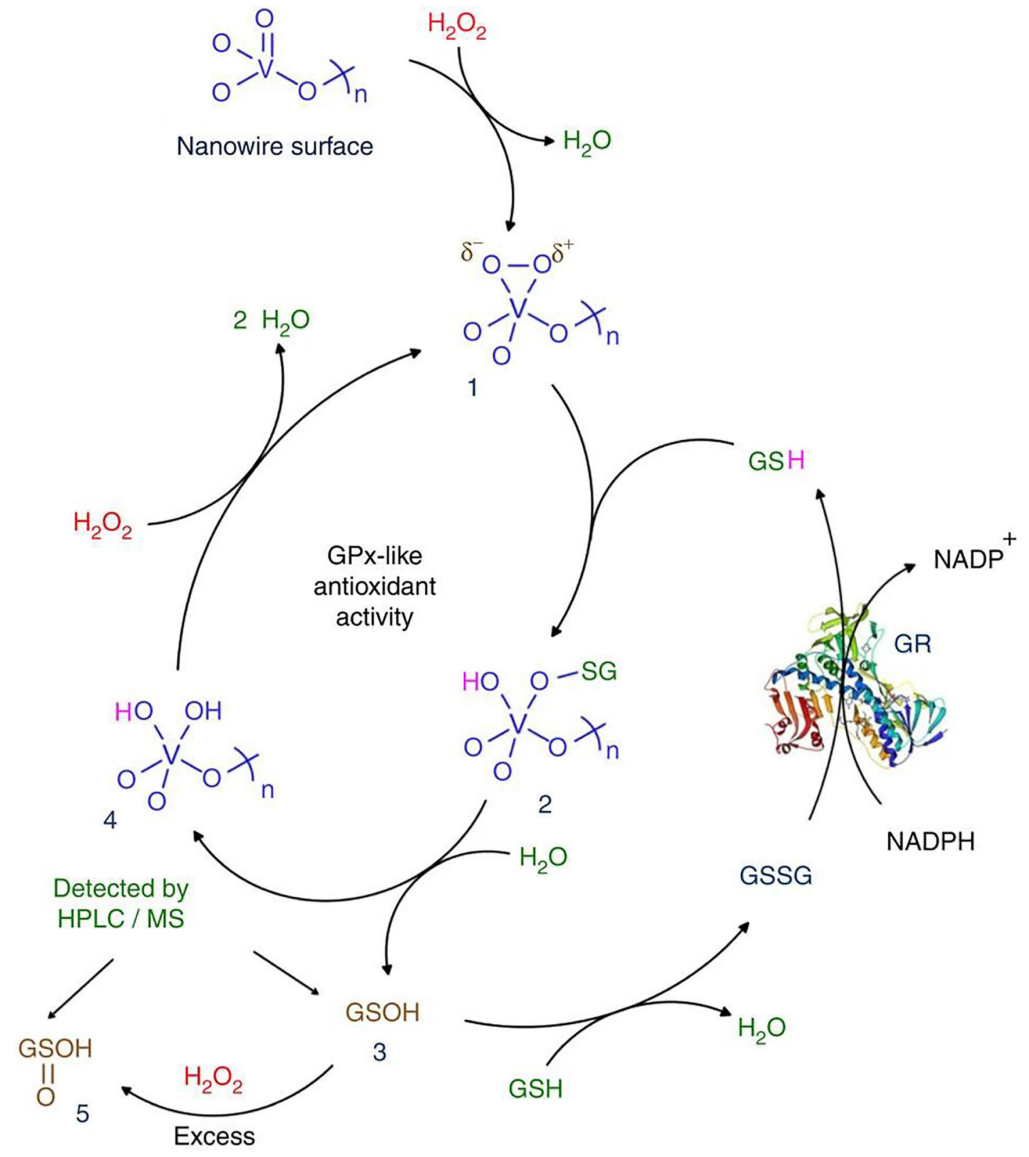
Mechanism of GPx activity of Vn [reproduced from Vernekar et al. (2014) with permission from the Springer Nature].

Mugesh's group confirmed that V_2_O_5_ nanowires (Vn) have GPx-like activity and biocompatibility and proposed a corresponding catalytic mechanism (Vernekar et al., 2014). In the nanostructure, the surface of Vn serves as a template for GSH reduction of H_2_O_2_. The functional groups on the surface of Vn changed to form vanadium peroxide species 1. The GS^−^ nucleophilically attacks the peroxide bond in complex 1, resulting in the formation of unstable sulfinate binding intermediate 2, which is hydrolyzed to produce glutathione sulfonic acid (3, GSOH) and dihydroxy intermediate 4. The hydrolysis of 2 to generate GSOH may be similar to the removal of HOBr from V-OBr intermediate in vanadium haloperoxidase (Natalio et al., 2012). Then, 4 reacts with H_2_O_2_ to produce peroxide compound 1. This is similar to one of the steps proposed in the mechanism of vanadium chloroperoxidase (Ligtenbarg et al., 2003). GSOH reacts with GSH to produce GSSG. GR/NADPH can reduce GSSG to GSH. It is worth noting that GSOH (3) is further oxidized to the corresponding sulfinic acid (5, GSO_2_H) under the condition of higher H_2_O_2_ concentration. In addition to GSH, other small thiol-containing molecules such as cysteine, cysteamine, and mercaptoethanol can also be used as thiol cofactors. In the presence of thiols, Vn showed notable thiol peroxidase activity through catalytic reduction of H_2_O_2_(Figure 2).

In the presence of peroxides, nanocarbon materials (CNMs) can catalyze the oxidation of organic substrates such as TMB (Shi et al., 2011; Zhang et al., 2013; Sun et al., 2015). Qu's group explored the dynamics of CO-COOH catalytic oxidation of TMB in the presence of H_2_O_2_. Based on the Michaelis Menten model, the kinetic parameters of GO-COOH, V_max_, and K_m_, were estimated and compared by using Lineweaver–Burk plots. At high H_2_O_2_ concentrations, the reaction catalyzed by GO-COOH was inhibited and followed ping-pong mechanism, which was similar to the reaction catalyzed by HRP. The UV spectra of GO-COOH showed that the addition of 88.2 mM H_2_O_2_ resulted in a red shift of about 19 nm, indicating the electron transfer from the top of the graphene valence band to the lowest unoccupied molecular orbital (LUMO) of H_2_O_2_ (Song et al., 2010).

## Enhance the Antioxidant Effect of Nanozymes

An ideal nano-antioxidant should have at least the following characteristics: (i) valid clearance of multiple primary and secondary reactive oxygen and nitrogen species (RONS); (ii) highly stable antioxidant activity against oxidative damage; (iii) the ability to prevent the activation of inflammation triggered by RONS; and (iv) excellent biocompatibility (Liu et al., 2017). Therefore, it is crucial to design the nanozyme reasonably to make it become an ideal antioxidant and widely used.

### Enhancing Nanozyme Activity

At present, the ROS scavenging capability of most nanozymes is moderate. Therefore, numerous strategies have been proposed to design more active nanozymes. One possible strategy is ion doping adding. For example, doping ions (such as Zr^4+^) into ceria NPs to modulate the ratio of Ce^3+^/Ce^4+^. The superoxide scavenging activity of ceria NPs could also be enhanced through an electron transfer strategy. Co-catalysis of various nanozymes can also improve catalytic activity.

#### Ion Doping Strategy

**Figure 3:**
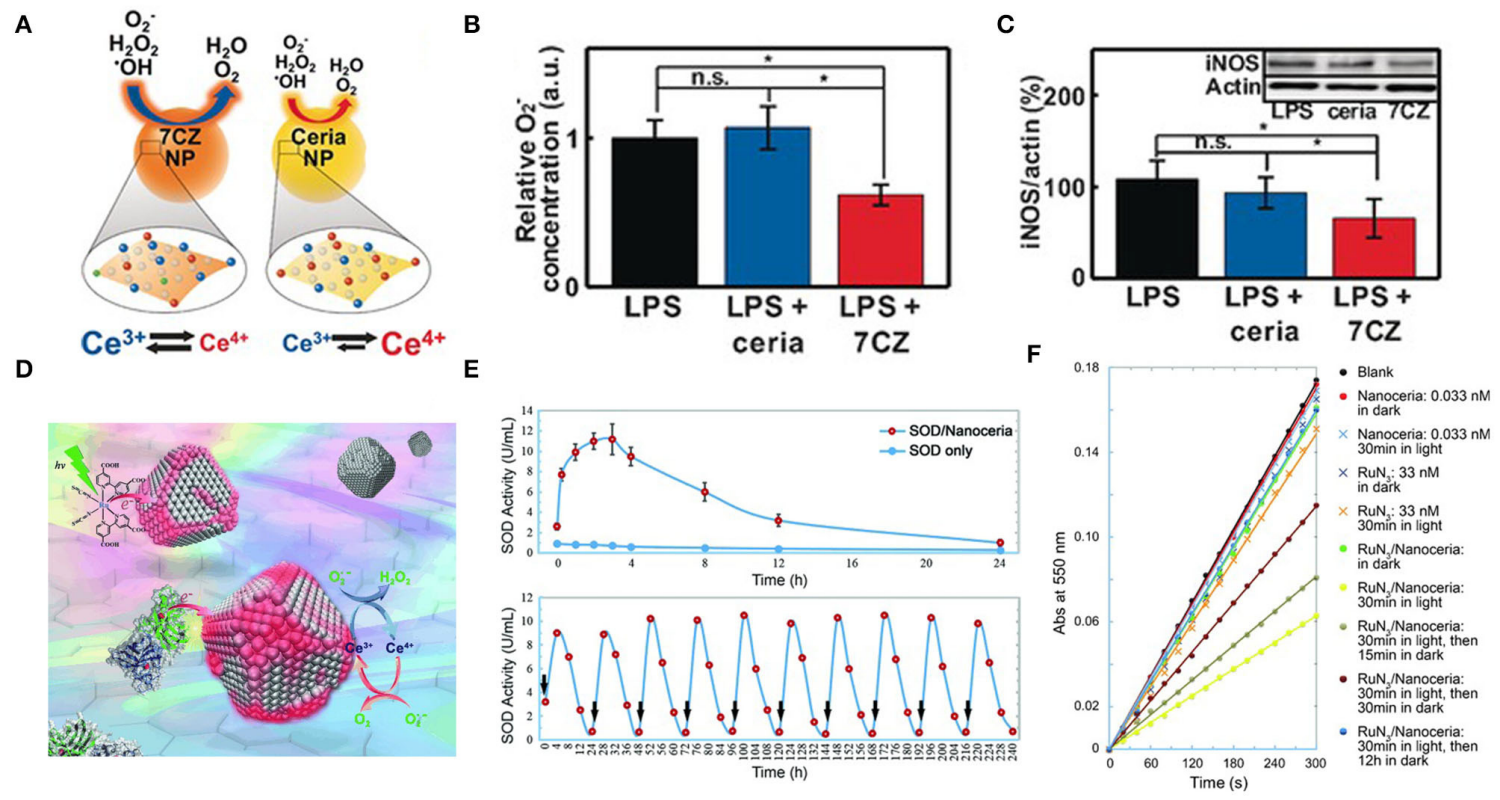
Ion-doping and electron transfer strategies enhance antioxidant enzyme activity. **(A)** Representing the different catalytic activities of ceria NPs and ceria-zirconia (CZ) NPs. **(B)** *in vitro* O_2_-assay: luminescence intensities of U937 cells were measured and expressed as relative O_2_-concentration, determined by measured values relative to those for the LPS-treated control; *n* = 4. **(C)** *in vitro* western blot analysis for iNOS: the blots were quantified using relative optical densities of iNOS and β-actin; *n* = 4 [reproduced from Soh et al. (2017) with permission from the John Wiley and Sons]. **(D)** Nanoceria acquire superoxide-scavenging ability after electron transfer. **(E)** The SOD mimetic activity of the CuZn-SOD/nanoceria mixture. **(F)** Effect of RuN_3_/nanoceria on superoxide anions from hypoxanthine/xanthine oxidase system as determined by a ferricytochrome C assay [reproduced from Li et al. (2014) with permission from the John Wiley and Sons].

Ceria nanoparticles (CeO_2_ NPs) are well-known as a superoxide dismutase (SOD) mimetic due to the redox cycle between Ce^3+^ and Ce^4+^. The surface Ce^3+^ to Ce^4+^ ratio is important because the capacity of removing ${\text{O}}_{2}^{•-}$ and ^•^OH is largely determined by the fractions of Ce^3+^(Baldim et al., 2018). Therefore, increasing the Ce^3+^/Ce^4+^ ratio is an effective strategy to improve the ability of nano-cerium to remove ROS. Seal's group doped the CeO_2_ NPs lattice with trivalent dopants (La, Sm, and Er) to significantly increase the surface Ce^3+^ ion concentration and produce a good SOD-like activity, as expected. Among the three dopants, Sm doped CeO_2_ NPs had the highest SOD activity, followed by La and Er doped CeO_2_ NPs. This ordering was due to the association energy of dopant atoms O-vacancies and the high concentration of Ce^3+^ on the surface. CeO_2_ NPs with higher content of Ce^3+^ were effective scavengers of intracellular ROS (Gupta et al., 2016). Hyeon's group reported ceria–zirconia nanoparticles [Ce_0.7_Zr_0.3_O_2_(7CZ)] in which the Zr^4+^ is used to modulate the ratio of Ce^3+^/Ce^4+^ (Soh et al., 2017). They demonstrated that the rate of Ce^4+^ to Ce^3+^ reduction is strongly increased throughout the Zr^4+^-containing NPs (Figure 3A). This finding may be due to the fact that the ionic radius of Zr^4+^ ion (0.084nm) is smaller than that of Ce^4+^ ion and Ce^3+^ ion (0.097 nm and 0.114 nm, respectively), which can reduce the lattice strain caused by the increase of the ionic radius of Ce^4+^ ion to Ce^3+^ ion. In *in vitro* lipopolysaccharide (LPS)-induced inflammation model, 7CZ NPs is more effective in scavenging ${\text{O}}_{2}^{-}$ compared to the ceria NPs (Figure 3B). In the inflammatory process, iNOS gene expression is upregulated and induced NO production (Grisham et al., 1999). Excessive NO can lead to vasodilation and hypotension, and eventually septic shock (Crawford et al., 2004). In LPS-stimulated macrophages, 7CZ NPs reduced NO and iNOS proteins, while ceria NPs did not show any effect (Figure 3C). Through the determination of extracellular lactate dehydrogenase content, 7CZ NPs could better inhibit the LPS-induced release of LDH, suggesting that it could provide better cell protection. These *in vitro* data indicated that compared to ceria NPs, 7CZ NPs are more effective in blocking the abnormal inflammatory response of macrophages by clearing the ROS/reactive nitrogen species (RNS) and other effects on the inflammatory pathway. Huang's group doped Mo into Pt_3_Ni nanocrystals to obtain highly active nanozymes, which are ~80 times the commercial catalyst activity (Huang et al., 2015). Zhang's group reported that a trimetallic (triM) nanozyme was obtained by doping transition metal molybdenum in platinum-palladium nanoparticles, which significantly improved the antioxidant activity (Mu et al., 2019b). The density functional theory (DFT) simulation results showed that the Mo atom has a strong attraction compared to the Pt and Pd atoms, which helps to separate the small unit into two atoms that are further apart and are independent of the free radical unit. Further analysis of the electrostatic potential (ESP) and the electron localization function (ELF) showed that Mo atoms are more attractive to small units and decrease the binding ability of adjacent Pt atoms. When small units are attached to triM nanozyme, there is a higher chance that they will be stretched by Mo and other atoms. Therefore, Mo doping can improve the catalytic efficiency of nanozymes.

#### Electronic Transfer Strategy

Another method to accelerate the induced reduction of Ce^4+^ to Ce^3+^ is the electron transfer strategy (Figure 3D). Zhang's group found that one of their ceria nanoparticles, which is (5.1 ± 0.4) nm in size, had an inappreciable Ce^3+^/Ce^4+^ ratio and weak SOD mimetic activity. But when co-incubated with CuZn-SOD in a PBS solution, the activity of ceria NPs was activated within a few minutes (Figure 3E). In the process of scavenging superoxide free radicals, there is a certain possibility that electrons are transferred from the Cu-Zn SOD to ceria NPs accompanied by the reduction of copper ions. Ceria NPs acts as an electronic sponge that can store electrons and regenerate active sites that scavenge superoxide radicals. Mixing [Ru (dcbpy)_2_ (NCS)_2_] with ceria NPs into PBS, they also found that the activity of SOD mimetic in the mixture increased significantly under visible light (Figure 3F), and gradually disappeared after the removal of light. These results suggested that the activity of ceria NPs could be excited after interfacial electron transfer (Li et al., 2014). Similarly, the bio-conjugation of ceria NPs with apoferritin exhibited an excellent ROS scavenging activity due to charge transfer at the interface between protein corona and ceria NPs, resulting in a change in the valence of the oxide and increasing the fraction of Ce^3+^ (Liu X. Y. et al., 2012). Qu's group reported a graphene oxide (GO)-Se nanozyme that exhibited higher GPx-like activity than SeNPs, possibly due to the large surface area and strong electron transfer ability of GO (Huang et al., 2017). Singh's group found that Fe_3_O_4_ NPs exhibited increased peroxidase-like activity in ATP presence over a wide range of pH values and temperatures. In terms of mechanism, it was found that ATP participated in the single-electron transfer reaction by complexing with Fe_3_O_4_ NPs, leading to the generation of hydroxyl radicals, which enhanced the activity of peroxidase-like at physiological pH (Vallabani et al., 2017).

#### Confinement Space

**Figure 4:**
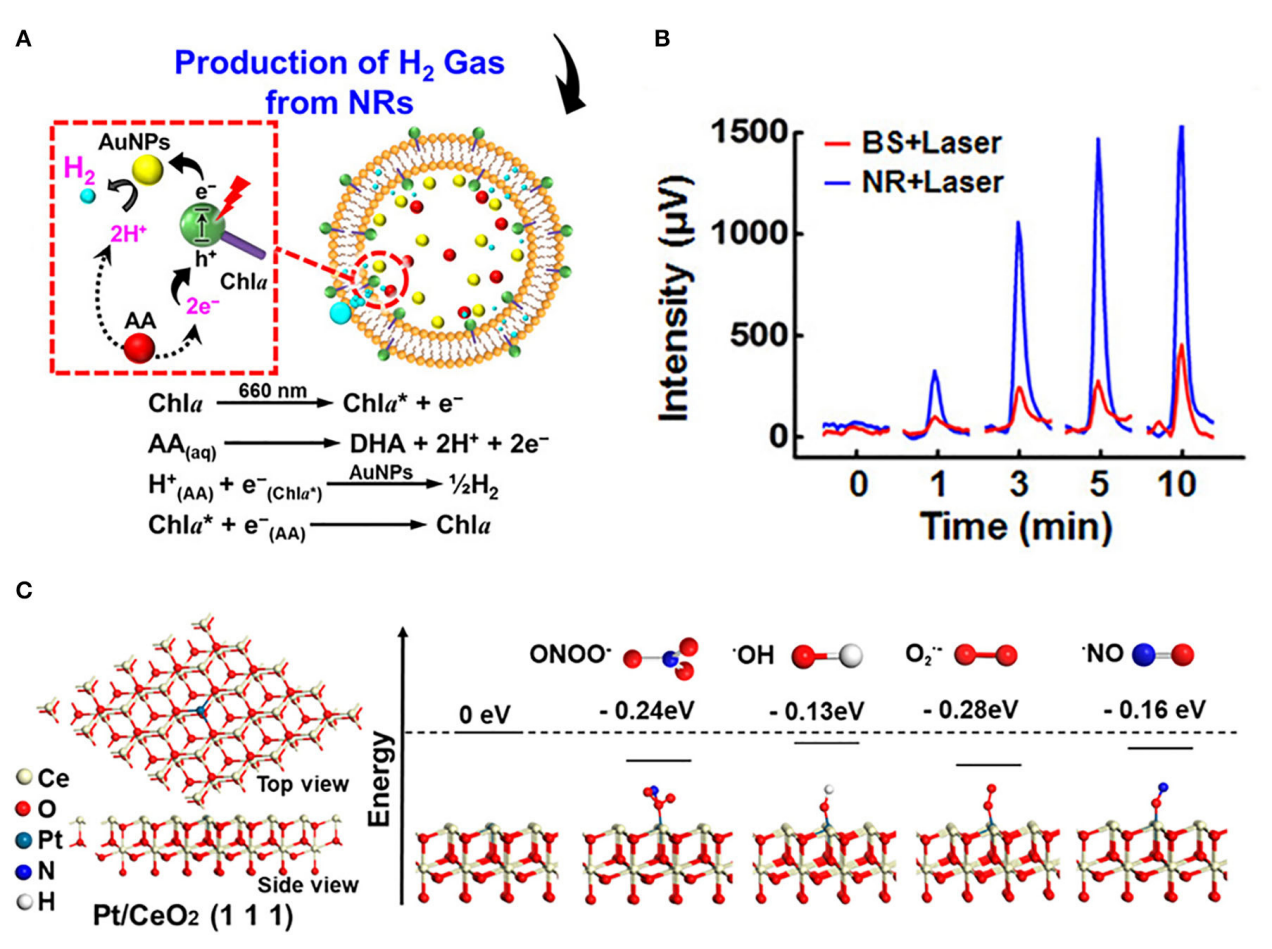
Introducing confinement space and single atom catalysis to enhance antioxidant activity. **(A)** Composition/structure of photodriven NR and mechanisms of its photosynthesis of H_2_ gas *in situ*. **(B)** Cumulative H_2_ concentrations generated in BS and NR following laser irradiation [reproduced from Wan et al. (2017) with permission from the American Chemical Society]. **(C)** Atomic structure of Pt/CeO_2_ along (111) facets and energy feature of the segment model with different radical units [reproduced from Yan et al. (2019) with permission from the American Chemical Society].

Nanoreactors provide a confining space in which reactive molecules and catalysts are encapsulated to enhance the activity of the reaction. The Au @ SiO2 yolk/shell structured nanoreactor framework was used for catalytic reduction of p-nitrophenol (Lee et al., 2008). Another nanoreactor composed of Pd @ meso-SiO_2_ showed excellent activity in the Suzuki coupling reaction (Chen et al., 2010). Amphiphilic networks have also been used as nanoreactors to stabilize and enhance enzyme catalytic activity in organic solvents (Bruns and Tiller, 2005). Nickel nanoparticles encapsulated by nitrogen-doped hollow carbon nanocapsules showed superior activity in oxygen reduction reaction. The low catalytic activity of the catalyst was compensated by increasing the collision rate in the nanocapsules. Defects in the graphene nano-shell allowed oxygen and hydroxyl to diffuse easily (Li B. et al., 2017). The CeO_X_ nanoparticles, encapsulated in hollow microspheres, can remove ROS more efficiently in a limited space (Ju et al., 2017). Sung's group encapsulated chlorophyll a, l-ascorbic acid, and gold nanoparticles in a liposome system to construct a multicomponent nanoreactor for efficient photo-driven hydrogen (H_2_) production (Figure 4A). The results of gas chromatography analysis showed that the cumulated H_2_ concentrations from the nanoreactors consistently surpassed those from bulk solution (Figure 4B) (Wan et al., 2017). This confinement space may raise the local concentration of the reaction, increase the probability of molecular collision, and thus improve the efficiency of the catalytic reaction.

#### Single Atom Catalysis

Single atom catalysis can be used as an essential tool to enhance catalytic activity. For example, Lee's group synthesized heme cofactor-resembling Fe–N_4_ single-site-embedded graphene in which the activity of each iron ion was 5 million times higher than that of Fe_3_O_4_ NPs (Kim et al., 2019b). Atom-dispersed metal centers can maximize the utilization efficiency of atoms and the density of active sites. Dong's group prepared the single-atom nanozymes of carbon nanoframe–confined axial N-coordinated single-atom Fe (FeN_5_ SA/CNF). The active sites of FeN_5_ SA/CNF were comparable to those of axially coordinated heme of natural redox enzyme. Taking oxidase catalysis as a model reaction, the theoretical calculations and experimental studies clearly showed that the highest oxidase-like of FeN_5_ SA/CNF is due to the synergistic effect and electron donation mechanism. It is worth noting that the oxidase-like activity of FeN_5_ SA/CNF was 17 times and 70 times higher than that of the square planar FeN_4_ catalyst and commercial Pt/C with normalized metal content, respectively (Huang et al., 2019). Ming's group anchored single-atom Pt on ultrasmall CeO_2_ nanoclusters, and the scavenging activity of RONS increased by 2–10 times compared with CeO_2_ clusters (Yan et al., 2019). The higher Ce^3+^/Ce^4+^ ratio in Pt/CeO_2_ and the catalytically active sites provided by single-atom Pt with oxygen vacancies are the two main reasons for improving the catalytic activity. Pt single atoms tend to be stabilized at CeO_2_ (111) and have a strong attraction, reducing the energy of units of free radicals bound to the surface of Pt/CeO_2_ (111) (Figure 4C), thereby enhancing the RONS scavenging ability. Zhao's group used Mn_3_[Co(CN)_6_]_2_ MOF as supporting material to frame a single atom of Ru with a load-weight ratio of up to 2.23 wt%. Ru partly replaced Co as a single atom catalytic site for endogenous oxygen production. Since the ligand terminal carbon had a stronger coordination ability with Ru than Co, partial substitution of Co was realized. The high catalase-like activity of this nanozyme should be attributed to six unsaturated Ru–C_6_ coordination sites, resulting in the rapid decomposition of H_2_O_2_ into O_2_ to overcome the tumor hypoxia (Wang et al., 2020). In addition, Lee's group developed N-and B-doped reduced graphene (NB-rGO) as a carbon nanomaterial mimicking peroxidase, whose catalytic activity was much higher than that of traditional carbon-based peroxidase-like nanozymes, even comparable to horseradish peroxidase (HRP). N- and B-codoping converts inert pyridine N atoms into catalytically active centers while retaining the peroxidase-like activity of a single B atom. Synergistic effect of N- and B-codoping further enhanced peroxidase activity over the undoped or single doped graphene (N or B) (Kim et al., 2019a).

#### Molecular Imprinting

Nanozymes are nanomaterials that mimic the activity of natural enzymes, and but most of them lack substrate specificity. The molecular imprinting technology of nanoenzymes provides a simple solution to this problem, but also improves the catalytic activity. Molecularly imprinted polymers (MIPs) are polymerized from monomers around template molecules (Schirhagl, 2014). The selected monomers are usually complementary to the properties of the template to form pre-polymer-bound complexes (Chen et al., 2011). During polymerization, the template is imprinted by the crosslinked polymer matrix. After removing the template, a cavity is generated for rebinding the template (Mahajan et al., 2019). The combination of PtPd nanoparticles with molecular imprinting enhanced peroxidase-like activity (Fan et al., 2017). Shen's group developed an efficient strategy for co-catalyzing peptide disulfide bond formation by molecularly imprinted polymer microzymes and inorganic magnetic nanozymes (Chen et al., 2017). Liu's group added acrylamide and nisopropylacrylamide (NIPAAm) as monomers and N, N′-methylenebis(acrylamide) (MBAAm) as a cross-linker to the Fe_3_O_4_ NPs and TMB (or ABTS) mixture. After adding the initiators into the system, the nanogels were obtained by precipitation polymerization. The substrate templates were then rinsed off to create binding pockets. The TMB and ABTS imprinted gels are named T-MIP and A-MIP, respectively. The authors next measured the rates of the nanozymes at various substrate concentrations to obtain enzyme parameters. The k_cat_ of T-MIP nanogel (15.0 s^−1^) is more than twice that of free Fe_3_O_4_ NPs and A-MIP when oxidizing TMB. For oxidizing ABTS, the A-MIP gel has the highest activity and affinity. T-MIP has 2.8-fold higher k_cat_/K_m_ (6.8 × 10^−2^ s^−1^ μM^−1^) than that of bare Fe_3_O_4_ (2.4 × 10^−2^ s^−1^ μM^−1^). When oxidizing ABTS, the same gel showed ~3 times lower k_cat_/K_m_ than bare Fe_3_O_4_. Similarly, the AMIP has 4-fold higher specificity than bare Fe_3_O_4_ for oxidizing ABTS but 1.5-fold lower for oxidizing TMB. Since TMB has a positive charge and ABTS has a negative charge, the imprinting was further improved by incorporating charged monomers. The TMB imprinted nanogels are named T-MIPneg if containing anionic AMPS and named T-MIPpos if containing cationic DMPA. The T-MIPneg has the best catalytic efficiency, 15-fold higher than that of the bare Fe_3_O_4_ NPs, much better than the 3-fold improvement for the T-MIP gel without the negative AMPS monomer. In the best case, the selectivity for TMB over ABTS using the T-MIPneg nanogel is 98-fold, while the selectivity for ABTS over TMB using the A-MIPpos is 33-fold (Zhang et al., 2017). Liu's group used surface science to understand the enhancement of activity by dissecting enzyme reactions to substrate adsorption, reaction, and product release. The enrichment of local substrate concentration induced by imprinting was about eight times, and the increase of substrate concentration could promote the improvement of the activity. The diffusion of the substrate on the imprinted gel layer was studied by pre-culture experiment, and the difference between the imprinted and non-imprinted gel layer was also highlighted. The activation energy of substrate imprinting sample was the lowest, 13.8 kJ/mol. The isothermal droplet calorimetry using the substrate and product samples imprinted separately showed that the product release rate was also improved after imprinting (Zhang et al., 2019).

#### Others

Choosing appropriate structure-oriented agents to synthesize nanoparticles may be an effective strategy to improve nanoparticles' catalytic activity. Kuang' group prepared a Cu_X_O-Ph nanocluster with phenylalanine (Phe) as a structure-directing agent with good biocompatibility and the properties of multiple antioxidants enzyme-like (Hao et al., 2019). The authors found that the selection of different molecular structure-oriented agents resulted in nanoclusters' different shapes and activity. The authors synthesized five other nanoclusters using five other amino acids as structure-directing agents, among which Cu_X_O -Ph showed the highest catalytic activity. Through the nitrogen adsorption test, it is found that the total volume and pore size of Cu_X_O-Phe are larger than those of other materials except Cu_X_O-Tyr. Although the size and surface area of CuXO-Phe (65 nm) is smaller than that of Cu_X_O-Tyr (186 nm), the catalytic activity of CuXO-Phe is much higher than that of Cu_X_O-Tyr, possibly because the ligand of the material plays an essential role in regulating its activity.

Single nanoparticle enzymes sometimes cannot achieve the desired antioxidant effect. The combined use of multiple nanoparticle enzymes can co-catalyze the elimination of ROS and perform a better scavenging effect. Qu's group assembled V_2_O_5_ and manganese dioxide (MnO_2_) nanoparticles through dopamine to construct a synergistic antioxidant system with multiple enzyme mimicking activities. V_2_O_5_ nanowires have GPx-like activity, and MnO_2_ nanoparticles are used to mimic SOD and CAT. The *in vitro* and *in vivo* experimental data showed that their biocompatible multi-nanozymes system had excellent intracellular ROS removal capacity, which protected intracellular components from oxidative damage, indicating its potential application in anti-inflammatory (Huang et al., 2016).

The activity can be activated by increasing the water solubility of the inorganic nanoparticles. Bulk Cu(OH)_2_ is highly water-insoluble (KL = 5.6·10^−20^ mol^3^·L^−3^) and does not exhibit any catalytic activity. However, after surface modification with glycine, it is easy to disperse in an aqueous medium, showing SOD-like activity that exceeds the activity of the natural CuZn enzyme (Korschelt et al., 2017).

### Regulating the Activity of Nanozymes

Some nanozymes have been found to have antagonistic peroxidase-like and catalase-like activities, such as Fe_3_O_4_ NPs, platinum NPs, and gold NPs. They were able to decompose H_2_O_2_ into toxic hydroxyl radicals (^•^OH) which are highly active and harmful to cells by the activity of peroxidase-like. In contrast, hydrogen peroxide could be broken down into H_2_O and O_2_ by the activity of catalase-like. The size, morphology, and surface catalytic sites of nanozymes have essential effects on their activity. Therefore, it is necessary to exert the antioxidant activity of nanozymes through reasonable regulation.

#### PH Regulation

**Figure 5:**
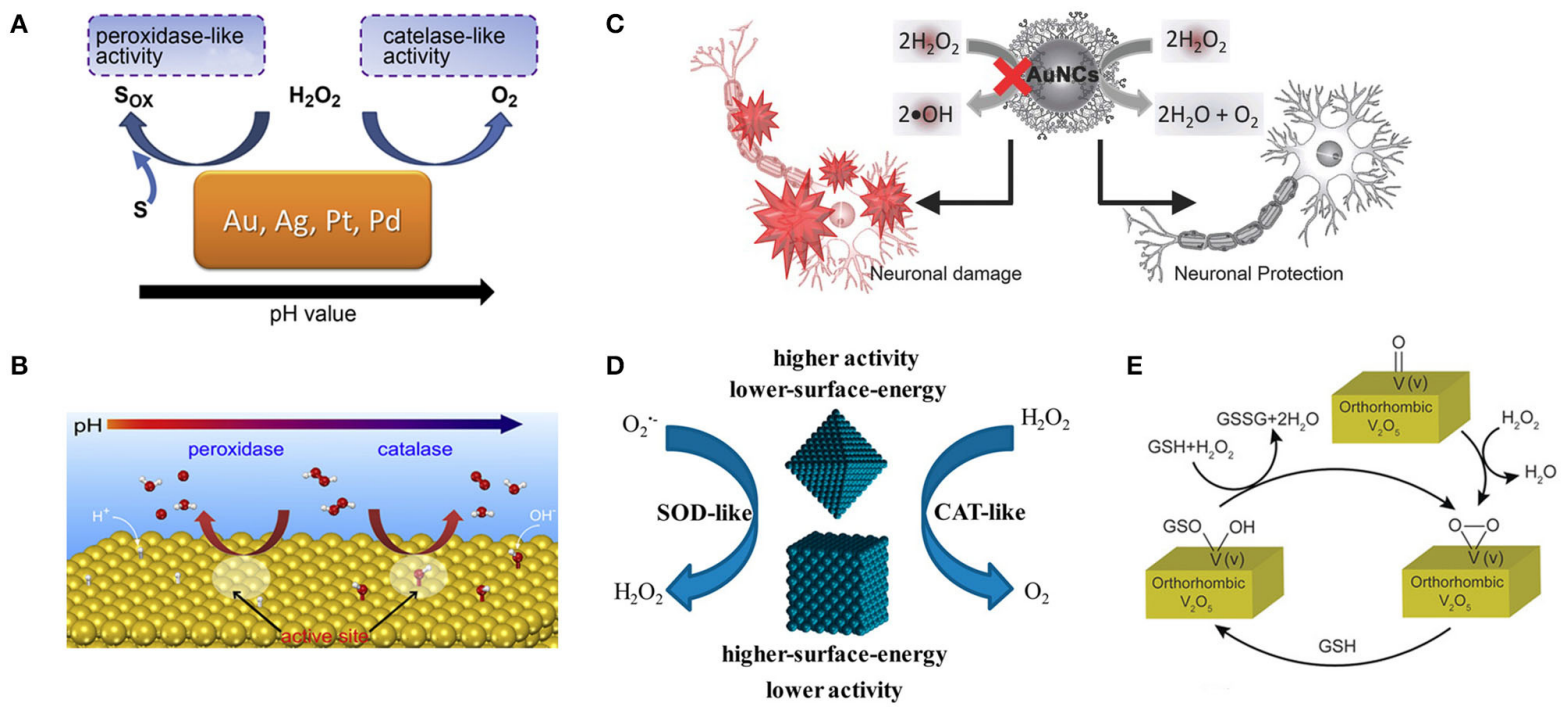
Various methods for regulating the activity of nanozymes include PH regulation, surface modification, and the effect of surface energy. **(A)** The pH-switchable peroxidase-like and catalase-like activities of Au, Ag, Pt, and Pd metals. **(B)** The predicted mechanisms for the pH-switchable peroxidase-like and catalase-like activities of the metals [reproduced from Li et al. (2015) with permission from the Elsevier]. **(C)** A schematic diagram shows the dendrimer-encapsulated AuNCs with the catalase-like activity to detoxify H_2_O_2_ for primary neuronal protection against oxidative damage [reproduced from Liu C.-P. et al. (2016) with permission from the John Wiley and Sons]. **(D)** Lower surface energy {111}-faceted Pd octahedrons have greater intrinsic antioxidant enzyme-like activity than higher surface energy {100}-faceted Pd nanocubes [reproduced from Ge et al. (2016) with permission from the American Chemical Society]. **(E)** Schematic representation of the reaction of H_2_O_2_ and GSH on the surface of orthorhombic V_2_O_5_ crystal [reproduced from Ghosh et al. (2018) with permission from the John Wiley and Sons].

The activities of various nanozymes have been found to have pH-adjustable properties, and exploring their intrinsic mechanism is of great significance for us to precisely regulate the activities of nanozymes. Yan's group reported that Fe_3_O_4_ magnetic nanoparticles (NPs) had an intrinsic peroxidase-like activity with a pH of 3.5 (Gao et al., 2007), and Gu's group reported that these particles had catalase-like activity under neutral pH conditions (Chen et al., 2012). Yin's group reported that Ag and Au NPs could catalyze the rapid decomposition of H_2_O_2_. At lower pH, the breakdown of H_2_O_2_ was accompanied by the production of ^•^OH, and at higher pH, by the production of O_2_. These particles also exhibited SOD-like activity when the pH = 7.4 (He et al., 2012, 2013). Gao's group studied the mechanism of PH-adjustable peroxidase and catalase-like activities of gold, silver, platinum, and palladium nanomaterials (Figure 5A) by calculation and experiment. The peroxidase-like activity of these metals at low pH is due to the basic-like decomposition of H_2_O_2_ on the metal surfaces. In contrast, the catalase-like activity at high pH is thought to be due to the acid-like decomposition of H_2_O_2_ on the metal surfaces (Figure 5B). They also proved that the activity of the enzyme is an inherent property of metals and has nothing to do with the environment. The relative enzymatic activity of metals with similar surface morphology can be predicted by the relative adsorption energy between H_2_O_2_ and metals (Li et al., 2015). The results are instructive for the design, synthesis, and application of metal-based artificial enzymes.

#### Surface Modification

Since the surface atoms are the critical catalytic sites, the antioxidant capacity of these nanoparticles can be fully exerted by choosing appropriate coating molecules to modify nanoparticles to block peroxidase-like activity and preserving catalase-like activity. Lin's group accidentally discovered that gold nanoparticles surface modified with amine-terminated PAMAM dendrimers lost peroxidase-like activity while still retained catalase-like activity under different pH conditions related to the biological microenvironment (Figure 5C). The authors proposed a possible mechanism that polymeric 3°-amines that are abundant on the surface of AuNCs-NH_2_ can be oxidized by hydroxyl radicals, which was confirmed by ^1^H NMR measurements. AuNCs-NH_2_ thus acquired the ability to scavenge ^•^OH and inhibit peroxidase-like activity (Liu C.-P. et al., 2016). However, with the gradual oxidation of tertiary amines, the peroxidase activity of nanoparticles is gradually restored, which may be detrimental to the long-term use of nanoparticles and broader application. Karakoti's group demonstrated that modifying the surface of CeO_2_ NPs with suitable ligands, especially triethyl phosphite (TEP) and tris(2,4,6 trimethoxy-phenyl)phosphine (TTMPP), could alter their redox properties and reverse the oxidation state. It was evident from XPS and PL studies that both TEP and TTMPP could electron-couple with the surface cerium ions of cerium CeO_2_ NPs. TEP can be used as a reductant to reduce the surface Ce^4+^ ion on CeO_2_ NPs, thus promoting the SOD activity of CeO_2_ NPs. It can be speculated that TTMPP is a large molecule, which can block the active surface sites of CeO_2−x_ NPs and make them lose SOD activity, but this does not explain how it activates their catalase activity. Therefore, a more comprehensive understanding of ligands' role in the regulation of enzyme-like activity of NPs is needed (Patel et al., 2018). Yan's group attached histidine to Fe_3_O_4_ NPs increasing its peroxidase-like activity by 20-fold (Fan et al., 2017). Liu's group adsorbed fluoride on nanoceria increasing its oxidase-like turnover by nearly 100-fold (Liu X. P. et al., 2016). Li's group prepared MIP around TiO_2_ photocatalyst also showing enhanced activity (Shen et al., 2009).

#### Control of Surface Energy

There is a tight correlation between the catalytic activity of metal-based nanomaterials and their surfaces. Different crystal forms of the same nanomaterial have different surface energies and may exhibit different catalytic activities. Yin's group found that the lower-surface-energy Pd octahedron {111} had higher antioxidant enzyme activity than the higher-surface-energy Pd nanoparticle {100} (Figure 5D). Their theoretical calculations showed that Pd octahedron with lower surface energy exhibits higher catalytic activity by lowering the reaction energy of scavenging reactive oxygen species, consistent with experimental observations. This study also provides a new perspective on the design of highly active antioxidant nanozymes (Ge et al., 2016). Mugesh's group synthesized four orthogonal V_2_O_5_ nanozymes with different morphologies [nanowires (VNw), nanosheets (VSh), nanoflowers (VNf), and nanospheres (VSp)] and found that their activity was independent of surface area. The differences in their GPX-like activity are due to differences in surface v-peroxide speciation rates (Figure 5E). In the same crystal system, the exposed crystal facets can adjust the H_2_O_2_ reduction capacity of V_2_O_5_ nanozyme. These results suggest that we can fine-tune the redox properties of nanomaterials by designing their surfaces (Ghosh et al., 2018).

### Enhancing Biocompatibility

It has been shown that some inorganic nanoparticles can interact with lipid, proteins, and DNA, thereby impairing the integrity of biofilms and the function of enzymes (Cedervall et al., 2007; Wang et al., 2008; Pelka et al., 2009), so improving the biocompatibility of nanoparticles is essential for their more extensive biological application.

Nanoparticles encapsulated in proteins can improve the biocompatibility of nanoparticles. Yeung group fixed BSA onto Au-Pt nanocomposites through electrostatic interaction, and the modified nanoparticles still maintained high antioxidant activity (Xiong et al., 2014). Knez's group encapsulated Pt NPs and PtAu NPs in apoferritin (Zhang et al., 2010, 2011), respectively, and Najaf's group reported apoferritin-encapsulated silver-gold nanoparticles (Dashtestani et al., 2018). The obtained nanoparticles not only have excellent biocompatibility but also can effectively remove ${\text{O}}_{2}^{•-}$ and H_2_O_2_. Apoferritin is a globular protein with an outer diameter of 12 nm and an inner cavity diameter of 7.6 nm. 14 small channels, 3–4 Å in diameter, run through the protein shell, providing size selection for ions and small molecules (Ford et al., 1984). These nanoparticles could safely enter human intestinal Caco-2 cells capable of expressing ferritin receptors constitutively without showing any toxicity to the cells. The protein shell of apoferritin can also avoid the aggregation of noble metal nanoparticles in solution and improve the stability of nanoparticles, which is beneficial to its long-term application in biological systems. The biocompatibility of nanoparticles can be improved by changing the surface stabilizer of nanoparticles. Erlichman's group reported that citrate/EDTA stabilized ceria NPs were well-tolerated and absorbed by the liver and spleen far less than previous nanoceria formulations when given intravenously to mice (Heckman et al., 2013). Pompa's group demonstrated that their citrate -capped Pt NPs did not exhibit significant cytotoxicity to cells *in vitro* (Moglianetti et al., 2016). PVP (Su et al., 2015) and PEG (Liu et al., 2017) are generally used polymer stabilizer because of their low cost, good water solubility, outstanding biocompatibility, and commercial availability.

Magnetite nanoparticles (Fe_3_O_4_ NPs) are proved to be biocompatible nanomaterials and have broad prospects in various biomedical applications (Lee et al., 2015). Fan's team found that the catalytic activity of Fe_3_O_4_ NP has a novel biocompatibility mechanism significantly different from that of conventional inert NP (Wang et al., 2018). The authors found that both nanoparticles induced response to oxidative stress by comparing the cellular effects of two ferric oxide nanoparticles (Fe_3_O_4_ and α-Fe_2_O_3_). Nevertheless, Fe_3_O_4_ NPs significantly delayed the production of toxic reactive oxygen species (ROS) and reduced autophagy and programmed cell death due to their antagonism of inherent catalase-like activity. The dynamic equilibrium mechanism proposed in this work inspires us to improve nanomaterials' biocompatibility by introducing antioxidant properties.

### Others

Enhancing the endogenous antioxidant ability of cells is an effective way to improve the ability of cells to resist oxidative stress. Interestingly, Alrokayan's group found that ceria NPs and Mo NPs could significantly increase in intracellular level of antioxidant molecule glutathione (GSH) in cells [human breast (MCF-7) and human fibrosarcoma (HT-1080)] challenged with oxidants (Akhtar et al., 2015a,b). This discovery can provide us a new perspective to explore the antioxidant mechanism of nanozyme, not only considering the antioxidant properties of nanozyme itself, but also taking the impact of nanozyme on biological environment into account.

The targeting properties of nanoparticles can be changed by surface modification of targeting agents or changing the size of nanoparticles. As the only biological barrier, the blood-brain barrier protects the brain from potentially harmful compounds in the blood, so, unfortunately, it prevents the build-up of nanoparticles in the brain (Hawkins and Thomas, 2005). During cerebral ischemia, the blood-brain barrier is damaged, leading to an increase in permeability (Kim et al., 2012; Jiang X. et al., 2018). Some nanoparticles can pass through the damaged part of the blood-brain barrier into the damaged part of the brain. However, the accumulation of nanoparticles in the brain is limited, which also limits the treatment of nanoparticles. Shi's group modified Angiopep-2 (ANG) onto CeO_2_ nanoparticles to create a BBB-targeted nanoplatform (Bao et al., 2018). ANG binds specifically to the over-expressed LDLR protein (LRP) in the cells that make up the BBB, significantly increasing the accumulation of nanoparticles in the brain through receptor-mediated endocytosis. Excess ROS, present in mitochondria, both intracellular and extracellular, plays a special role in disease (Kwon et al., 2018). Hyeon's group prepared three kinds of ceria NPs by changing the size and surface modification, which can selectively eliminate these three kinds of ROS. Small-sized nano-cerium is easily absorbed by cells, eliminating ROS from the cytoplasm. Triphenylphosphonium-modified nano-cerium can enter mitochondria and remove ROS in mitochondria. Because the endocytosis of large nanoparticles is suppressed, the 300 nm-sized cluster-ceria NPs remain outside the cell and eliminate the extracellular ROS (Kwon et al., 2018).

## Biological Application of Antioxidant Nanozymes

### Anti-aging

As animals age, oxidative damage accumulates in their bodies, which is closely related to aging, behavioral decline, geriatric disease, and lifespan (Finkel and Holbrook., 2000). Dugan's group administered carboxy fullerenes SOD mimetics to non-transgenic, and non-senescence accelerated mice starting at middle age. The authors found that this chronic treatment reduced age-related oxidative stress and the production of mitochondrial free radicals, significantly prolonged lifespan, and improved the performance of mice in Morris water maze learning and memory tasks (Quick et al., 2008). A mixture of Pd and Pt nanoparticles has been reported to reduce age-related skin atrophy in mice. Drosophila melanogaster has been widely used to study and explain the mechanism of some complex biological processes, including development, metabolism, and aging (Shibuya et al., 2014). Song's group found that dietary Fe_3_O_4_ NPs significantly reduced ROS levels in aged drosophilae, boosting their ability to climb and prolonging their lifespan (Zhang et al., 2015).

### Cytoprotection

**Figure 6:**
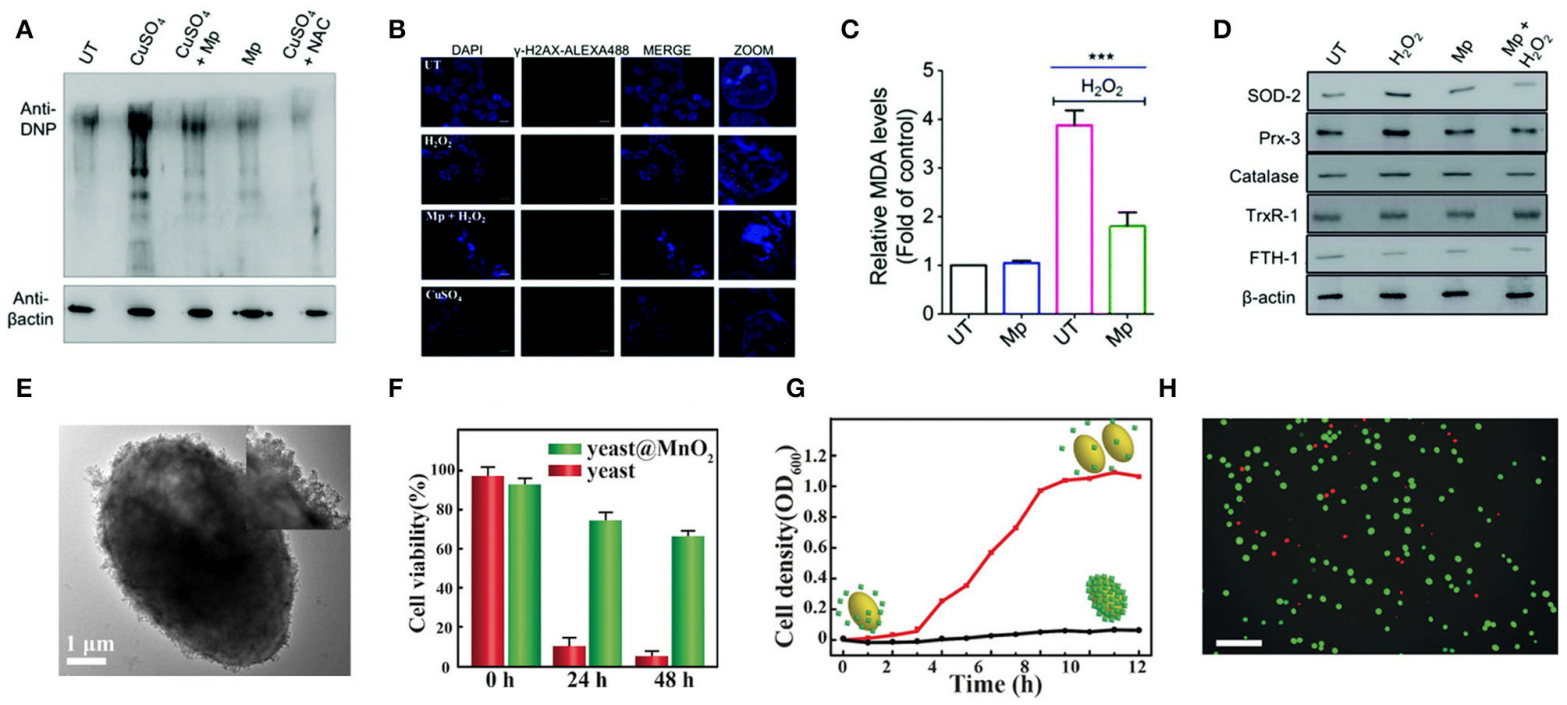
Cellular protection of nanozymes. **(A)** HEK293T cells subjected to treatment were lysed and derivatized using 2,4-DNPH (2,4-dinitrophenylhydrazine) before SDS-PAGE. Immunoblot analysis was performed using anti-DNP antibodies. N-Acetyl cysteine treated cells (NAC) (100 mM) were used as the positive control. **(B)** Immunofluorescence microscopy was carried out to analyze the amount of DNA double-strand break by detecting the formation of γH2AX foci formed through phosphorylation. **(C)** The level of MDA formed in the cell lysate was quantified to determine the extent of lipid peroxidation using the TBARS assay. **(D)** The expression of Nrf2 responsive genes and antioxidant machinery was investigated in the presence of Mp by western blotting using specific antibodies for various stress-markers and antioxidant enzymes [reproduced from Singh et al. (2019) with permission from the Royal Society of Chemistry (RSC)]. **(E)** TEM image of yeast@MnO_2_. Inset: HR-TEM image. **(F)** Cell viability of native yeast and yeast@MnO_2_ after incubation with H_2_O_2_ for different time. **(G)** Growth curve of yeast@MnO2 with (red line) and without (black line) glutathione (GSH) stimuli. **(H)** Live/dead stained cells after removal of MnO_2_ shells. Scale bar: 50 μm. [reproduced from Li W. et al. (2017) with permission from the John Wiley and Sons].

Excessive production of intracellular ROS and inefficiency of the endogenous antioxidant system can cause oxidative stress, which leads to cell component damage and cell apoptosis. Nanozymes with antioxidant capacity can protect cells from oxidative stress. For example, Mn_3_O_4_ NPs can mimic three major antioxidant enzymes, including SOD, GPx, and CAT (Singh et al., 2017). Mugesh's group explored specific ways in which Mn_3_O_4_ NPs protect cells from oxidative damage, and demonstrated that the NPs do not interfere with the endogenous antioxidant mechanisms of cells (Singh et al., 2019). Their experimental data proved that this nanozyme could prevent ROS from damaging proteins (Figure 6A), breaking DNA double-strand (Figure 6B), and lipid peroxidation in cells (Figure 6C). This work also ascertained that under conditions of oxidative stress, this nanozyme would not affect the expression of Nrf2 protein (Figure 6D), which is a crucial regulator of the expression of antioxidant proteins in cells. This study demonstrated the remarkable ability of nanoenzymes to regulate cellular oxidation-reduction homeostasis without interfering with intracellular antioxidant proteins/enzymes.

Qu's group proposed a novel cell protection strategy, which uses manganese dioxide (MnO_2_) nanozyme as a smart shell to encapsulate a single living cell to achieve long-term protection and operation (Li W. et al., 2017). The authors encapsulated the yeast cells in the MnO_2_ shell through the process of biomineralization (Figure 6E), which enhanced the cell's tolerance to severe physical stress, such as dehydration and lyase, and improved the survival time of the cells under high levels of toxic chemicals (Figure 6F). What is more, once the shell is removed by stimulation with pure biomolecule glutathione (GSH), these encapsulated cells can fully restore growth and function (Figures 6G,H). Stabler's group prepared a ceria NPs- alginate composite hydrogel, which could play a useful role in protecting the encapsulated cells (Weaver and Stabler, 2015).

### Inflammation Treatment

Excessive ROS induces microglia polarization, from the anti-inflammatory M2 phenotype to the pro-inflammatory M1 phenotype (Zhang et al., 2016). This phenotypic change usually aggravates neuronal damage and M1-activated microglia produce more ROS through up-regulated anaerobic glycolysis (Orihuela et al., 2016). Li's group demonstrated that CeNP-PEG effectively protected neurons by blocking the inflammatory signaling pathway triggered by ROS, allowing the phenotype of microglia to switch from proinflammatory M1 to anti-inflammatory M2 (Zeng et al., 2018). A large amount of toxic reactive active oxygen (ROS) in cigarette smoke (such as superoxide radicals, hydrogen peroxide, hydroxyl radicals) will increase the oxidative stress in the lungs of cigarette smokers, causing severe pulmonary inflammation, and leading to serious lung diseases (Stämpfli and Anderson, 2009). Effective removal of ROS from cigarette smoke is very important to prevent smoking-induced inflammatory lung diseases. Nagai's group reported a Platinum nanoparticle stabilized with polyacrylate (Pt-PAA) which can efficiently quench ROS. In *in vitro* and *in vivo* experiments, the results suggested that the PT-PAA inhibited cell death and pulmonary inflammation in smoking mice (Onizawa et al., 2009).

**Figure 7:**
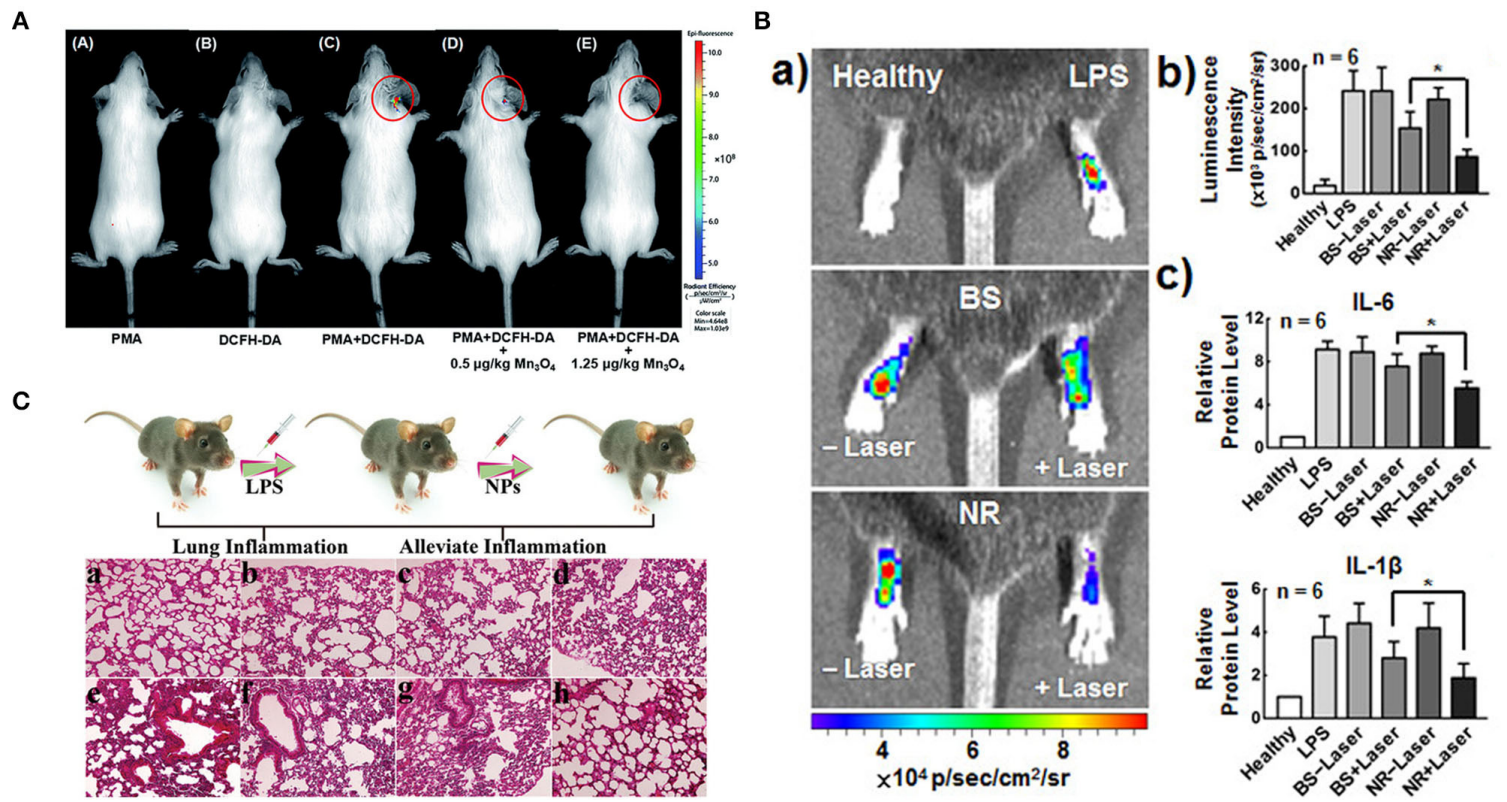
Therapeutic effects of nanozymes on different inflammatory models. **(A)** *in vivo* fluorescence imaging of mice with PMA-induced ear inflammation [reproduced from Yao et al. (2018) with permission from the Royal Society of Chemistry (RSC)]. **(B)** (a) IVIS images and (b) corresponding L-012 luminescence intensities of ROS in LPS-induced inflamed paws following treatment with BS and NR without/with laser irradiation. (c) Levels of inflammatory cytokines IL-6 and IL-1β [reproduced from Wan et al. (2017) with permission from the American Chemical Society]. **(C)** Schematic illustration of lung inflammation model, and histological images of mouse lung tissue with different treatments: (a) untreated; (b) pDA; (c) Se; (d) Se@pDA; (e) LPS; (f) pDA and LPS; (g) Se and LPS; (h) Se@pDA and LPS [reproduced from Huang et al. (2018) with permission from the John Wiley and Sons].

Wei's group reported a CuTA nanozyme by the coordination of Cu^2+^ and tannic acid, which was utilized to mimic antioxidative enzymes including SOD-like activity, catalase-like and ^•^OH elimination capacity. These characteristics endued CuTA nanozyme with an excellent ROS scavenging ability. The nanozyme was further applied in cigarette filter modification to reduce the damage caused by ROS in cigarette smoke to mouse models (Lin et al., 2019). Their group also reported another Mn_3_O_4_ nanozyme which was synthesized via a hydrothermal method (Yao et al., 2018). They demonstrated that the nanozyme possessed extraordinary SOD mimicking activities due to the mixed oxidation valence states of Mn^2+^ and Mn^3+^. In addition, the Mn_3_O_4_ nanozyme also possessed CAT mimicking activity and hydroxyl radical scavenging activity. Fluorescence images and corresponding fluorescent intensity indicated that the dose-dependent intracellular ROS scavenging activities of Mn_3_O_4_ NPs by using Hela cell line as a model. In an ear inflammation mouse model, experiment data showed that the Mn_3_O_4_ NPs possessed efficient ROS scavenging capacity and negligible toxicity toward live tissues (Figure 7A).

Sung's group reported a multicomponent nanoreactor (NR) that comprises chlorophyll a (Chla), l-ascorbic acid (AA), and gold nanoparticles that are encapsulated in a liposomal (Lip) system that can produce H_2_ gas *in situ* upon photon absorption to mitigate inflammatory responses (Wan et al., 2017). Chla, a photosensitizer, is excited (Chla^*^) by absorbing light with wavelengths of 660 nm and an electron-hole pair is thus generated. The hole in the excited Chla^*^ can accept a new electron from AA which as an electron donor, returning to its ground state. Colloidal AuNPs, a catalyst, can promote conversion of the electrons from the excited Chal^*^ and the protons from the oxidized AA to H_2_ gas. H_2_ can selectively reduce ^•^OH to H_2_O while retaining other required ROS for normal signal regulation. In LPS-induced mouse paw inflammation model, the ROS, ir-6, and IL-1β levels in the inflammatory tissues irradiated with NR laser were lower (Figure 7B). The multicomponent system had excellent anti-inflammation effects and great potential in mitigating tissue inflammation. Selenium-based nanozymes have also been reported to remove ROS because of the special electronegativity of selenium, which gives it unique chemical properties, such as redox reactivity (Li F. et al., 2017). Xu's group reported selenium-doped carbon quantum dots, which had redox-dependent reversible fluorescence property, were effective in protecting cells from oxidative stress (Li F. et al., 2017). Qu's group reported a Se@pDA nanocomposite, in which selenium possessed excellent GPx-like activity, and dopamine had reducibility, which could synergistically remove harmful ROS from cell components (Huang et al., 2018). In the LPS-induced pneumonia model in mice, this nanocomposite could significantly alleviate the inflammatory response, including nuclear contraction, inflammatory cell infiltration, thickening of the alveolar wall, and protect life systems from oxidative damage (Figure 7C). Melanin nanoparticles have been reported to have multiple antioxidant and anti-inflammatory properties *in vitro* (Liu et al., 2017). In a rat model of ischemic stroke, the authors evaluated the *in vivo* efficacy of the nanozyme by pre-injection into the lateral ventricle. Compared with the saline control group, the PEG-MeNPs pre-treated rats had smaller infarct areas, significantly reduced sensitivity to ischemia, and significantly inhibited the formation of ${\text{O}}_{2}^{•-}$. The evaluation results of *in vivo* toxicity indicated that nanozymes did not induce systemic cytokine responses in mice and showed excellent blood compatibility. These results all suggested the great potential of PEG-MeNPs to prevent oxidative damage to the ischemic brain. Zhang's group reported that the PtPdMo triM nanozymes could exhibit the best antioxidant activity under a neutral environment, effectively scavenging ROS and RNS (Mu et al., 2019b). *In vitro* experiments showed that the nanoparticles protected the H_2_O_2_ -and LPS- treated neutral cells from oxidative damage. All *in vivo* toxicity evaluation results showed that triM nanozymes did not produce severe inflammation and immune reactions in the body and were a relatively safe antioxidant. Besides, the elimination of overproduced free radicals after treatment with nanoenzymes reduced neuroinflammation and significantly improved survival rate and reference memory in mice.

### Wound Repair

The recovery of tissue integrity and tissue function of injured skin is crucial to wound repair and regeneration, but the synergistic effect of both is still challenging to achieve (Wu et al., 2018). At present, most wound healing treatment focuses on the process of structural restoration (Ghobril and Grinstaff, 2015). However, little work has been done on the microenvironment regulation of the injured site by the intrinsic regeneration ability of the host (Forbes and Rosenthal, 2014). Due to the increased production of ROS in the injured area, which may induce a series of harmful effects such as cell senescence (Finkel and Holbrook, 2000), fibrosis scar (Pellicoro et al., 2014), and inflammatory reaction (Mittal et al., 2014), it is suggested that the reduction of oxidative stress in the microenvironment of the injured area will help to promote the healing of regenerated wounds. Water-soluble CeO_2_ nanoparticles can penetrate wound tissue, reduce oxidative damage to cell membrane and protein, and accelerate the healing of full-thickness dermal wounds (Chigurupati et al., 2013). However, the hydrophilic coating may weaken their tissue adhesion properties and affect the effect of wound repair. Ling's group fixed ultrasmall CeO_2_ nanocrystals on the surface of uniform mesoporous silica nanoparticles (MSN) and prepared a multipurpose ROS-scavenging tissue adhesive nanocomposite (Wu et al., 2018). CeO_2_ nanocrystals loaded with MSN not only have muscular tissue adhesion strength but also much limit the damage mediated by ROS, which effectively accelerates wound healing. More importantly, the wound area manifested an unexpected regenerative healing characteristic, marked by skin attachment morphogenesis and the formation of limited scarring. This strategy is also suitable for the repair of wounds that have a great need for the removal of ROS and tissue adhesion.

### Cancer Treatment

Radiotherapy (RT) is one of the primary methods of cancer treatment. However, inadequate intratumoral radiation energy deposition and hypoxia-related radiation resistance are still the biggest obstacles to RT. Manganese dioxide (MnO_2_) nanoparticles, which can decompose hydrogen peroxide into oxygen due to their inherent CAT-like activity, have been used to enhance RT *in vivo* (Song et al., 2016). However, after the interaction of MnO_2_ NPs and x-rays, the radiation dose cannot be increased by effectively emitting electron radiation. Choi's group used porous platinum nanoparticles as a new nanomedical platform to solve two obstacles that restrict the efficacy of RT. *In vivo* experiments showed that high atom-number platinum interacted with tumor X-rays could emit electron radiation effectively, maximally enhance the radiation dose of tumor cells, and the porous PtNPs could rapidly convert H_2_O_2_ to O_2_, overcoming the microenvironment of tumor hypoxia by utilizing its high porosity and large surface area (Li et al., 2019).

### Treatment of Traumatic Brain Injuries

Traumatic brain injury (TBIs) causes many complications, the most prominent of which is nerve inflammation (Russo and McGavern, 2016). Reactive oxygen species (ROS) and reactive nitrogen species (RNS), especially RNS caused by inflammation can cause continuous damage to TBI, which can lead to severe tissue necrosis and apoptosis (Russo and McGavern, 2016). The RNS after TBI is highly active and toxic and difficult to remove (Simon et al., 2017). Hu's group reported a carbogenic nanozyme which was prepared by simple microwave heating with lysine and ascorbic acid and exhibited an ultrahigh ROS (including ${\text{O}}_{2}^{•-}$, H_2_O_2_, and ^•^OH) and RNS (such as ^•^NO and ONOO^−^) scavenging efficiency (~16 times higher than AA) (Hao et al., 2019). After the LPS- and H_2_O_2_-damaged neuron cells were treated with carbogenic nanozyme, the cell viability was significantly improved by eliminating various RONS. After the nanozyme was injected intravenously into the TBI mouse model, as the injection time increased, the BBB permeability and the MMP-9 expression level gradually decreased, indicating that the nanozyme could effectively repair the BBB destruction and subsequent brain edema. Furthermore, the spatial learning and memory capabilities of TBI mice could be effectively restored by the nanozyme treatment (Hao et al., 2019). This work confirmed the enormous potential of carbogenic nanozyme in the treatment of acute TBI. Ming's group used single-atom Pt/CeO_2_ bandage for local non-invasive treatment of TBI. Throughout the 30 days of treatment, the nanozyme bandages showed good stability and catalytic activity without any significant decline. After the nanozyme-based bandages were applied to the injured brain area of mice with TBI, the size and area of wounds were significantly reduced to normal levels. At the same time, the untreated group recovered only partially (~50%) (Yan et al., 2019).

### Treatment of Neurological Diseases

**Figure 8:**
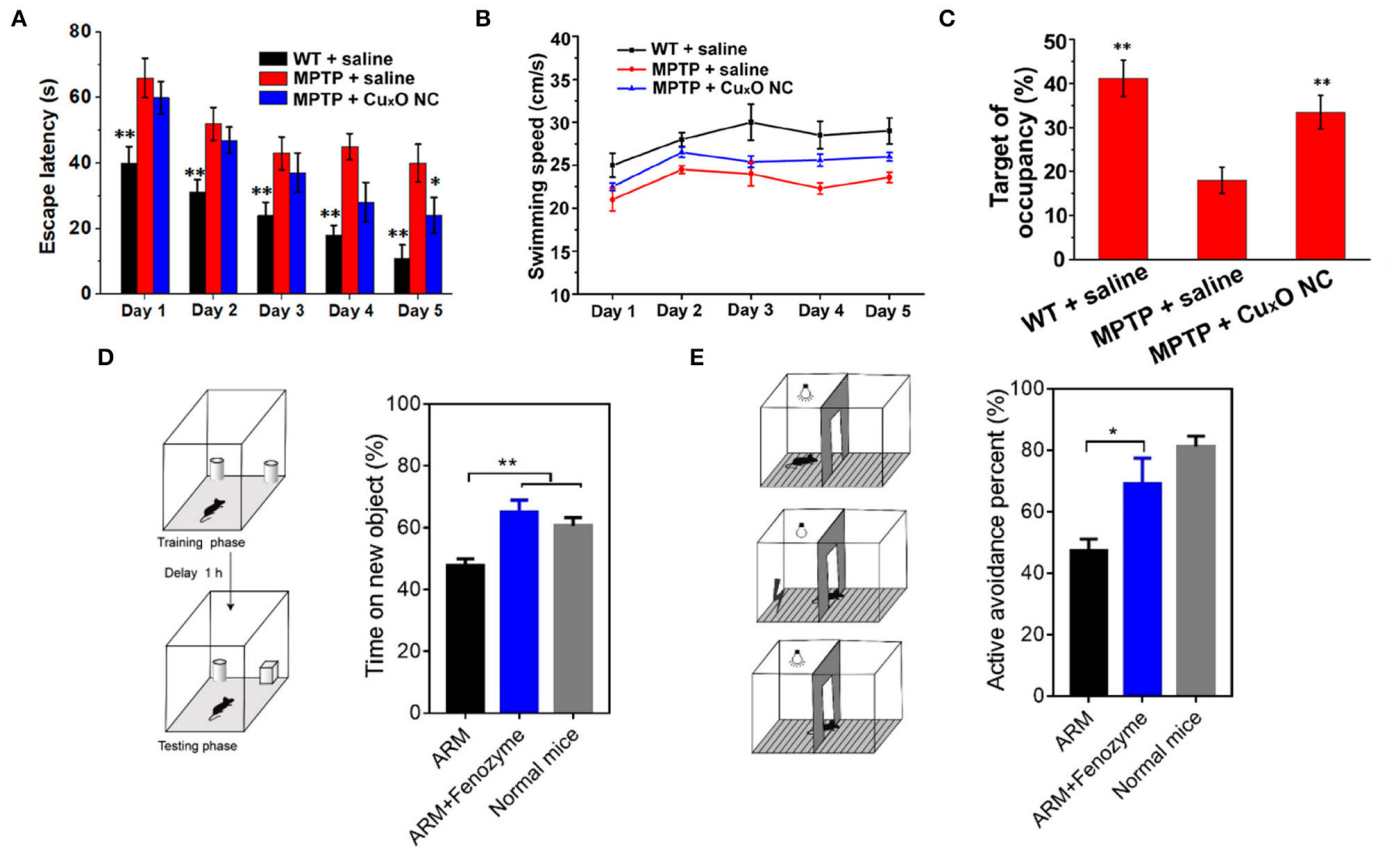
Treatment of nervous system diseases with nanozymes **(A)** Escape latency. **(B)** swimming speed. **(C)** relative time spent on the target quadrant where the escape platform used to be located [reproduced from Hao et al. (2019) with permission from the American Chemical Society]. **(D)** Time spent on the new object by ARM- and ARM + Fenozyme-treated ECM mice (*n* = 5). **(E)** Active avoidance percent by ARM- and ARM + Fenozyme-treated ECM mice in the last 30 times after 500 times of training (*n* = 5). Normal mice without infection were also used as a control [reproduced from Zhao et al. (2019) with permission from the American Chemical Society].

The Cu_X_O nanoparticle clusters can effectively inhibit the neurotoxicity of Parkinson's disease cell model and repair memory loss in Parkinson's disease mice (Hao et al., 2019). The PD group had less time in the target quadrant than the other group, and the motion paths were random, which reflected the memory impairment caused by MPTP. The mice treated with Cu_X_O -NCs showed spatially oriented swimming behavior and stayed in the target quadrant for a long time (Figures 8A–C), and the motor pathways were mainly concentrated in the target quadrant, suggesting that the Cu_X_O-NCs treatment repaired memory loss in PD mice.

Cerebral malaria is a deadly complication of malaria infection accompanied by severe central nervous system dysfunction. Traditional combined antimalarial therapy can not treat it effectively (Zhao et al., 2019). In experiments based on brain malaria mouse models, it was found that when encephalopathy occurs, microvascular endothelial cells are often damaged, and the blood-brain barrier is destroyed (Coban et al., 2018). Studies have shown that the rupture of an infected parasite releases free hemoglobin, which may lead to excessive production of ROS, damaging endothelial cells, and the blood-brain barrier (Pamplona et al., 2007). Therefore, ROS might be an essential regulator of damage to the blood-brain barrier during the occurrence of cerebral malaria. Based on the pathogenesis of malignant cerebral malaria, fan's group developed a ferritin nanozyme (Fenozyme), which consisted of recombinant human ferritin (HFn) protein shell that specifically targeted the BBB endothelial cells and the core of the Fe_3_O_4_ nanozyme, which had ROS scavenging activity. In the experimental cerebral malaria (ECM) mouse model, by injecting fenozyme, the damage of blood-brain barrier induced by parasites was well-inhibited, the survival rate of infected mice was improved, and the macrophages in the liver were polarized into M1 phenotype, and the clearance of malaria parasites in blood was promoted. Besides, fenozyme significantly reduced encephalitis and memory impairment in ECM mice treated with artemether (Figures 8D,E). These results indicated that ROS played an essential role in the development of cerebral malaria and that fenozyme combined with antimalarial drugs was a very effective treatment strategy (Zhao et al., 2019).

Amyloid-β peptide (Aβ) agglomeration plays an essential role in the pathogenesis of Alzheimer's disease (AD) (Hamley, 2012), accompanied by excessive ROS production (Cimini et al., 2012). CeO_2_ nanoparticles combined with enzymes or molecules resisting Aβ-aggregation have been used in the treatment of AD, showing a good therapeutic effect (Li et al., 2013; Guan et al., 2016). However, these nanoparticles lack a certain degree of targeting. Qu's group combined Aβ-target peptide KLVFF and C_60_ with up-conversion nanoparticles to construct a nano-platform for the treatment of AD (Du et al., 2018). In near-infrared light, Aβ-targeting nano-platform produced ROS that caused photooxidation of Aβ, which inhibited Aβ-aggregation and attenuated subsequent cytotoxicity. In the dark, the nanocomposite relieved oxidative stress by eliminating the overproduction of ROS.

### Others

Nanozymes can be used as an adjuvant to endow some nanostructures with antioxidant properties, thus arousing the potential of nanoparticles in biological applications. Komatsu's team reported an artificial O_2_ carrier with antioxidant activity, which is formed by combining hemoglobin-albumin clusters with Pt nanoparticles [Hb-HSA_3_(PtNP)]. The resulting nanocluster has a robust ability to bind oxygen and avoids damage from ${\text{O}}_{2}^{•-}$ and H_2_O_2_. In many clinical cases involving ischemia-reperfusion injury, such nanoclusters have high medical value and can be used as a substitute for red blood cells for blood transfusion (Hosaka et al., 2014). Qu's group developed a novel artificial metalloenzyme-based enzyme replacement therapy for the treatment of hyperuricemia. Uric acid enzyme (UA) and platinum nanoparticles (PtNPs) were tightly packed in the pores of mesoporous silica nanoparticles to form a tandem catalysis system. PtNPs could effectively eliminate H_2_O_2_ produced by UA, which enhanced the mammalian cell viability and had a significant therapeutic effect on hyperuricemia mice (Liu X. P. et al., 2016). CeO_X_ nanoparticles can effectively remove the ROS generated by ZnO during ultraviolet irradiation, thus providing broad-spectrum ultraviolet protection (Ju et al., 2017). Qin's group introduced PtNPs into the single-channel volumetric bar-chart chip (V-Chip) to detect biomarkers quantitatively (Song et al., 2014). To detect lung cancer biomarker CYFRA 21-1, they used a typical three-component sandwich ELISA method in which PtNPs were combined with probe antibodies and reacted with hydrogen peroxide to generate oxygen. At the same time, V-Chip can quantitatively measure the volume of oxygen generated. They used the PtV-Chip to assess the expression levels of HER2 in three breast cancer cell lines, suggesting that the PtV-Chip chip could be used to analyze biomarkers. Ceria NPs were introduced into bioabsorbable electronic stents to remove ROS produced in the perfusion by percutaneous coronary intervention and reduce inflammation that can cause thrombosis in the stent (Son et al., 2015).

## Conclusion and Outlook

In this article, we have gathered recent research on the design and development of novel antioxidants based on nanozymes. We summarized several approaches to enhance the antioxidant activity of these enzymes, including enhancing catalytic activity, regulating the exertion of catalytic activity, improving biocompatibility, and targeting, and stimulating intracellular antioxidant activity. Nanozymes are broadly used in the field of biomedicine as an antioxidant, such as anti-aging, cell protection, anti-inflammatory, wound repair, cancer treatment, traumatic brain injury, and neurological diseases.

Although considerable progress has been made, there are still some obstacles to be overcome. (1) Compared with natural enzymes, nanozymes have poorer selectivity for substrates and have no specific structure to bind to substrates. Although it can be improved by surface modification, shape modification, etc., it will also affect the activity of the nanozyme. Moreover, under some conditions, nanozymes will combine with various substrates, and some side reactions will occur, which is not conducive to application in the biological field. Therefore, it is very important to improve the ability of nanozymes to specifically bind to substrates. (2) Many of the catalytic mechanisms of nanozymes lack detailed theoretical research. A further understanding of the catalytic mechanism can help us better comprehend the relationship between the structure of nanozymes and catalytic performance, thereby better regulating catalytic efficiency and substrate selectivity. Eventually, the research of nanozymes will be transferred from empirical science to substantial and basic theoretical science. (3) Different synthesis methods and conditions will also affect the antioxidant properties of nanozymes. Therefore, more uncomplicated, programmable strategies for synthesis and fabrication are required, which may be beneficial for future research on nanozymes. (4) Evaluation of the toxicity of nanozymes is still at a short-term level, and accurate data on the distribution, metabolism, clearance, and antioxidant activity of nanozymes in organisms are lacking. Most biological applications remain at the stage of mouse studies, making it difficult to transition to clinical studies and actual production. Thus, more detailed biological data, such as the long-term toxicity, pharmacodynamics, pharmacokinetics, immunogenicity, and catalytic activity of nanozymes *in vivo*, are needed to reduce the distance from basic research to clinical application. (5) There is no uniform standard to analyze the antioxidant activity of nanoparticles. It will be beneficial to the research and development of this field to establish a unified standardized analysis method.

In short, nanozymes may play a vital role in the biomedical field as an ideal antioxidant in the foreseeable future. (1) The influence of the structure of nanozyme on its catalytic mechanism will be more apparent in the future. In this regard, optimal synthesis methods should be designed and developed through experimental and computational approaches to achieve the highest possible effect. (2) The types of nanozymes will further increase, for example, analogs of active centers can be created in natural enzymes and then incorporated into MOFs or other nanomaterials to mimic catalytic activity. (3) Standard methods for the determination of catalytic activity and kinetics of peroxidase-like nanozymes have been reported (Jiang B. et al., 2018). Uniform standards for analyzing the antioxidant activity of nanoparticles will soon be established, which will lead to more reliable results and significantly promote research in this field. (4) In addition to catalysis, nanomaterials also endow nanozymes with more functions, including magnetic, optical, and thermal properties. This multi-functional antioxidant nanozyme will enhance antioxidant activity and broaden its application fields. (5) With the further development of pathology, the pathogenesis of various diseases will be more explicit, which will be more conducive to the broader application of nanozymes.

## Author Contributions

RT wrote the first draft. JX, QL, CH, and JL modified the manuscript content and format. All authors contributed to the article and approved the submitted version.

## Conflict of Interest

The authors declare that the research was conducted in the absence of any commercial or financial relationships that could be construed as a potential conflict of interest.
